# The Japan Society for Surgical Infection: guidelines for the prevention, detection, and management of gastroenterological surgical site infection, 2018

**DOI:** 10.1007/s00595-020-02181-6

**Published:** 2020-12-15

**Authors:** Hiroki Ohge, Toshihiko Mayumi, Seiji Haji, Yuichi Kitagawa, Masahiro Kobayashi, Motomu Kobayashi, Toru Mizuguchi, Yasuhiko Mohri, Fumie Sakamoto, Junzo Shimizu, Katsunori Suzuki, Motoi Uchino, Chizuru Yamashita, Masahiro Yoshida, Koichi Hirata, Yoshinobu Sumiyama, Shinya Kusachi, The Committee for Gastroenterological Surgical Site Infection Guidelines, the Japan Society for Surgical Infection

**Affiliations:** 1 Department of Infectious Diseases, Hiroshima University Hospital, Hiroshima, Japan; 2 Department of Emergency Medicine, School of Medicine, University of Occupational and Environmental Health, Fukuoka, Japan; 3 Department of Surgery, Soseikai General Hospital, Kyoto, Japan; 4 Department of Infection Control, National Center for Geriatrics and Gerontology, Aichi, Japan; 5 Laboratory of Clinical Pharmacokinetics, School of Pharmacy, Kitasato University, Tokyo, Japan; 6 Perioperative Management Center, Department of Anesthesiology and Resuscitology, Okayama University Hospital, Okayama, Japan; 7 Division of Surgical Science, Department of Nursing, Sapporo Medical University, Sapporo, Japan; 8 Department of Surgery, Mie Prefectural General Medical Center, Mie, Japan; 9 Infection Control Division, Quality Improvement Center, St. Luke’s International Hospital, Tokyo, Japan; 10 Department of Surgery, Toyonaka Municipal Hospital, Osaka, Japan; 11 Division of Infection Control and Prevention, University of Occupational and Environmental Health, Fukuoka, Japan; 12 Division of Inflammatory Bowel Disease Surgery, Department of Gastroenterological Surgery, Hyogo College of Medicine, Hyogo, Japan; 13 Department of Anesthesiology and Critical Care Medicine, Fujita Health University School of Medicine, Aichi, Japan; 14 Department of Hepato-Biliary-Pancreatic and Gastrointestinal Surgery, International University of Health and Welfare, School of Medicine, Chiba, Japan; 15 JR Sapporo Hospital, Sapporo, Japan; 16 Toho University, Tokyo, Japan; 17 Department of Surgery, Tohokamagaya Hospital, Chiba, Japan

**Keywords:** Surgical site infection, Guidelines, Antibiotics, Drain

## Abstract

**Background:**

The guidelines for the prevention, detection, and management of gastroenterological surgical site infections (SSIs) were published in Japanese by the Japan Society for Surgical Infection in 2018. This is a summary of these guidelines for medical professionals worldwide.

**Methods:**

We conducted a systematic review and comprehensive evaluation of the evidence for diagnosis and treatment of gastroenterological SSIs, based on the concepts of the Grading of Recommendations Assessment, Development and Evaluation (GRADE) system. The strength of recommendations was graded and voted using the Delphi method and the nominal group technique. Modifications were made to the guidelines in response to feedback from the general public and relevant medical societies.

**Results:**

There were 44 questions prepared in seven subject areas, for which 51 recommendations were made. The seven subject areas were: definition and etiology, diagnosis, preoperative management, prophylactic antibiotics, intraoperative management, perioperative management, and wound management. According to the GRADE system, we evaluated the body of evidence for each clinical question. Based on the results of the meta-analysis, recommendations were graded using the Delphi method to generate useful information. The final version of the recommendations was published in 2018, in Japanese.

**Conclusions:**

The Japanese Guidelines for the prevention, detection, and management of gastroenterological SSI were published in 2018 to provide useful information for clinicians and improve the clinical outcome of patients.

**Electronic supplementary material:**

The online version of this article (10.1007/s00595-020-02181-6) contains supplementary material, which is available to authorized users.

## Introduction

The guidelines for the prevention, detection, and management of gastroenterological surgical site infections (SSIs) were published in 2018, in Japanese, by the Japan Society for Surgical Infection^(1)^. This is a summary of these guidelines in English for medical professionals around the world.

## Purpose of the guidelines

SSIs are the main focus of the Japan Society for Surgical Infection. The Society conducted three randomized controlled trials, on total gastrectomy, hepatectomy, and colectomy, to evaluate the duration of perioperative preventive antibiotic administration^(1)^. Several SSI guidelines have been revised or created; however, these are international guidelines and clinical situations differ among countries^(2−4)^. Therefore, we constructed the Japanese Guidelines for the prevention, detection, and management of gastroenterological SSIs to inform medical staff and improve patients’ outcomes.

## Methods used to construct the guidelines

The Committee for Gastroenterological Surgical Site Infection Guidelines within the Japan Society for Surgical Infection was formed in April, 2016. After studying the preparation methods for the guidelines and the Grading of Recommendations Assessment, Development and Evaluation (GRADE) system, the members devised Clinical Questions (CQ) and performed a systematic review using keywords in PubMed and Japana Centra Revuo Medicina Web (Ichushi-Web) between January, 2000 and March, 2016. Table 1 presents the findings of evidence assessed by following the procedures^(2–4)^. The strength of recommendations was graded with reference to the quality of the evidence, the preferences of the patient, the risks and benefits, and cost estimates. In terms of consensus-building, a vote was taken using the Delphi method and the nominal group technique (NGT), and issues with a support rate of more than 70% were approved. Table 2 shows the grading of the recommendations.

**Table 1:** Quality of evidence^(5)^

Comprehensive assessment of publications by outcomes and design
(1) Initial assessment: assessment by each study design group A: SR (systematic review), MA (meta-analysis), RCT (randomized controlled trial) C: OS (observational study) D: CS (case series, case report)
(2) Assessment of the presence/absence of factors that decrease evidence levels Risk of bias in study quality Inconsistent results (different conclusions in various papers) Indirect evidence (inconsistency between content within a paper and the CQ, or content in a paper which is not directly applicable to clinical use) Inaccurate data (insufficient number of cases) High probability of publication bias (only favorable results reported)
(3) Assessment of the presence/absence of factors that increase evidence levels Profound effects with no confounders (profound effects expected for all cases) Dose–response gradient (more profound effects expected with increased dosage) Possible confounders that diminish actual effects
Comprehensive assessment: overall quality of evidence across outcomes was assessed and graded as A (high), B (moderate), C (low), or D (very low)

**Table 2:** Strength of recommendations

Strength of recommendations	Contents
Consensus	Self-evident truth, ethically impossible to perform a high quality clinical study
1	Strongly recommended to perform
2a	Recommended to perform Evidence is moderate or strong, although evidence of effectiveness is sparse
2b	Evidence is sparse, but may be considered in the decision to perform Effectiveness can possibly be expected
3	Scientific evidence is not sufficient, so clear recommendation cannot be made Evidence is not sufficient to support or deny effectiveness
4	Recommended not to perform Harm or risk can possibly exceed benefit
5	Strongly recommended not to perform There is evidence to deny effectiveness (to show harm)

The proposal for the guidelines received comments from the public and feedback from members of the Japan Society for Surgical Infection, the Japan Surgical Society, the Japanese Society of Gastroenterology Surgery, the Japanese Society of Hepato-Biliary-Pancreatic Surgery, the Japanese Association for Infectious Disease, and Takeo Nakayama of the Department of Health Informatics, Kyoto University, School of Public Health. The guidelines were modified in response to this feedback and finally published in 2018. Table 3 lists the key recommendations.

**Table 3:** Summary of recommendations

1. Definition, epidemiology, and risk factors of SSI
(1) Surgical site infection is defined as an infection that involves the skin and subcutaneous tissue of the incision (superficial incisional) and/or the deep soft tissue (for example, fascia, muscle) of the incision (deep incisional) and/or any part of the anatomy other than the incision that was opened or manipulated during an operation (organ/space)
(2) The incidence of surgical site infection after gastrointestinal surgery was 9.6% according to the Japan Nosocomial Infections surveillance. The incidence of SSI is highest after esophageal surgery, followed by rectal surgery and hepatobiliary surgery (3) The risk factors for surgical site infection are ASA, wound class, prolonged operation time, diabetes, obesity, hyponutritional status, history of smoking, and intraoperative blood transfusion
2. Diagnosis criteria, surveillance, and causal bacterium of SSI
(1) The criteria suggested by the Centers for Disease Control and Prevention (CDC)/National Healthcare Saftety Netowrk (NHSN) are used for the diagnosis of SSI. In Japan, some of these criteria have been modified slightly (2) Some reports suggest that the incidence of SSI after gastrointestinal surgery decreases with surveillance. Surveillance is necessary to assess the true incidence of SSI (D, Consensus) (3) Surveillance for more than 30 days after surgery is necessary, including for discharged patients (C, Consensus). It is preferable that the surveillance include many examination methods such as bacterial culture in combination with surveillance by an infection control team (ICT) for evaluation (D, Consensus) (4) In Japan, a surveillance system such as JANIS and JHAIS report the latest detection methods. Reference to these data for each surgical procedure is recommended 3. Preoperative management of SSI (1) In digestive surgery, the incidence of SSI in patients with known nasal carriage of S. aureus may be high (2) Preoperative decolonization may be useful for preventing SSI in patients who are known nasal carriers of *S. aureus* (C, 2a). However, universal decolonization without screening is not recommended, to prevent the spread of resistance (B, 4). Candidacy for screening of *S. aureus* carriage should be determined based on the local epidemiology in the hospital, the patient’s risk factors for *S. aureus* infection, and the surgical procedure to be performed (3) Although it may be desirable to give effective antibiotic prophylaxis to patients carrying resistant bacteria, there is no clear foundation to recommend it (D, 3) (4) Since patients with preoperative malnutrition who undergo digestive operations have a high incidence of SSI, the committee recommends that the malnutrition status should be improved before surgery (B, 2a) (5) It is not effective to administer enhanced nutritional formulas before surgery for the purpose of preventing SSI in non-malnourished patients who undergo digestive operations (B, 3) (6) Preoperative smoking is a high-risk factor for SSI (B). Patients who discontinue smoking for 1 month before surgery may decrease their risk of SSI (C, 2a) (7) Preoperative regular alcohol consumption is a risk factor for SSI (C). The effectiveness of abstinence from alcohol to prevent SSI is not clearly indicated, but we suggest preoperative abstinence (D, 2b) (8) Long-term or high-dose steroids are risk factors for SSI (C). The administration of immunomodulators and biologics before surgery is not a risk factor for SSI (C). However, the effect of reducing these drugs on SSI incidence has not been studied. Reduction or withdrawal of these drugs should be planned based on the original disease (D) (9) Preoperative mechanical bowel preparation (MBP) alone does not appear to have a preventive effect on SSI (A). However, MBP with oral antibiotics added (OAMBP) is recommended since it may have a preventative effect on SSI (B, 2a) (10) Preoperative cleansing of the skin with chlorhexidine gluconate has no effect on preventing SSI (B, 4) (11) It has been recommended to shave to prevent SSI, and not to do so (A, 5). There is no difference in the incidence of SSI between clipper hair removal, no hair removal, or using hair depilation cream (B) 4. Prophylactic antibiotics (1) Treatment with prophylactic antibiotics is considered beneficial in gastrointestinal surgery because of its effectiveness in the prevention of SSIs after laparoscopic cholecystectomy (A, 2a) and inguinal hernia surgery (B, 2a) (2) Although evidence is limited, administration within 60 min before the surgical incision is preferred (D, 2b) (3) No high-quality studies have shown that the intraoperative re-administration of prophylactic antibiotics reduces SSI incidence, and the utility of re-administration is not known. Therefore, there is no basis for recommending when re-administration is appropriate (C) (4) In patients undergoing elective gastrectomy for gastric cancer when prophylactic antibiotics were administered only before surgery (including patients given additional intraoperative treatment when surgery exceeded 3 h), there was no increase in SSI incidence compared with those who also received prophylactic antibiotic treatment after surgery. For this reason, only administration before surgery (including additional intraoperative treatment when surgery exceeds 3 h) is recommended (B, 2a). Evidence of the duration of prophylactic antibiotic treatment in elective colectomy for colorectal cancer is limited, and at this point, the difference in the benefit of administration only before surgery (including additional intraoperative treatment when surgery exceeds 3 h) and administration both before and after surgery is unknown (C, 3). Note that this analysis focuses mainly on laparotomy data, and laparoscopic surgery is a topic for future investigation 5. Intraoperative management (1) Surgical hand scrubbing and rubbing exhibit are equally effective for SSI prevention. Either method is acceptable but should be performed appropriately (A, no recommendation) (2) The panel recommends alcohol-based antiseptic solutions with chlorhexidine gluconate for surgical site skin preparation for patients undergoing gastrointestinal surgical procedures (B, 2a) (3) The effectiveness of adhesive drapes in preventing SSI is unclear (C, 3) (4) The use of wound protector devices, especially the double-ring wound protector device used in gastrointestinal surgical procedures, reduces the rate of SSI (A, 2a) (5) We suggest the use of double-gloving during surgery to address safety concerns since glove perforation may cause occupational exposures, injuries, or infections (A, 2b) (6) The value of changing instruments during surgery for preventing SSI is unclear due to the lack of evidence; hence, we do not actively recommend this practice. However, it is recommended to change instruments to avoid the use of potentially dirty or contaminated surgical operations (D, 2b) (7) We recommend the use of antimicrobial-coated sutures for preventing SSI during digestive surgery (B, 2a) (8) We recommend wound irrigation, especially with high pressure, for preventing SSI (C, 2a). However, we cannot provide a recommendation for wound irrigation with disinfectant, antibiotics, or electrolyzed acidic aqueous solution due to the lack of evidence (D, 3) (9) We do not recommend peritoneal lavage for preventing SSI due to the lack of evidence (D, 3) (10-1) Drain placement after surgery for gastric cancer did not show any benefit for SSI prevention. Drain placement is not necessary because mortality and complication rates are also low (B, 3) (10-2) Complications, SSI incidence and mortality are similar with or without drains after laparoscopic cholecystectomy, but the operation time was shortened with non-drainage. Therefore, drain placement is not required (A, 4) (10-3) The absence of a drain after hepatectomy without biliary reconstruction tends to have a lower SSI rate, less ascites, and shorter hospitalization. Therefore, the committee recommended that drain placement after hepatectomy was unnecessary. (A, 4) (10-4) SSI after pancreatoduodenectomy tends to be higher in the no-drain group, and some studies have been discontinued due to increased mortality rates, so it is recommended to use a drain (B, 2b). As far as duration of the drain placement, the committee recommended that it be removed early according to the criteria for postoperative pancreatic juice and that patients should be selected carefully (10-5) No drain is preferable after appendectomy for preventing SSI. Drain placement may increase the incidence of complications and mortality. Therefore, we do not recommend drain placement after appendectomy. (B, 4) (10-6) In colon surgery, drain placement is unnecessary in the prevention of SSI (A, 4). The clinical benefit of the drain placement is unclear, and it might be unnecessary. On the other hand, drain placement could be considered in specific cases when it might contribute to reducing severe complications (A, 3) (10-7) Although subcutaneous drain placement may reduce the incidence of SSI, it is necessary to consider the indications for appropriate cases, methods, and duration. (B, 3) (11-1) Subcutaneous suturing using absorbed materials is recommended. (B, 3) (11-2) Continuous sutures tended to result in less wound dehiscence and fewer wound infections than interrupted sutures for subcutaneous suturing after gastroenterological surgery. Therefore, continuous sutures rather than interrupted sutures are recommended for subcutaneous suturing (B, 2a). In fascia closure, incidences of SSI and wound hernia did not differ between continuous sutures and interrupted sutures. Therefore, either method can be used (B, 3) (11-3) Subcutaneous sutures using absorbable materials do not reduce the incidence of SSI versus skin closure using a stapler. However, it is recommended for cosmetic purposes and patient satisfaction (B, 2b) (11-4) The rates of SSI and wound dehiscence associated with bioadhesives for primary wound closure were comparable to those of sutures alone. Bioadhesives may improve cosmetic results and shorten operation time in primary wound closure after laparoscopic surgery (C, 3) 6. Perioperative management (1) Implementation of an early recovery program is recommended to reduce the incidence of SSI in patients undergoing digestive surgery, as well as for shortening the length of hospital stay and accelerating the recovery of gut function (A, 2a). However, it remains unclear which components of the program are optimal for SSI prevention in various types of digestive surgery (2) Preoperative carbohydrate loading does not prevent SSI after digestive surgery. Therefore, implementation of preoperative carbohydrate loading prevention is not recommended for SSI prevention (A, 3) (3) A blood glucose level of less than 150 mg/dL is desirable because strict blood glucose control during the postoperative period reduces the incidence of SSI significantly in digestive surgery patients with and those without diabetes mellitus (B, 2b). On the other hand, blood glucose should be monitored closely because of the inherent risk of a hypoglycemic event (4) There are no guidelines on whether perioperative oral hygiene contributes to the prevention of SSI in gastrointestinal surgery because of the lack of evidence (D). On the other hand, perioperative oral care may help to prevent postoperative pneumonia after esophagectomy (5) Intraoperative warming for maintaining normothermia is recommended for SSI prevention. (B, 2a) (6) High oxygen concentrations (FIO_2_ of 0.8) during and within 2–6 h after colorectal surgery may reduce the risk of SSI (B, 3). However, high concentrations of oxygen also have adverse effects such as absorption atelectasis and oxygen toxicity. Furthermore, the safety of high oxygen concentrations during long operations is not supported. The indication for high FIO_2_ should be evaluated carefully (7) Although early postoperative oral and enteral feeding does not reduce the risk of SSI (B), it is recommended because of other benefits such as shortening the hospital stay 7. Wound management (1) It is preferable to use protective wound dressings for relatively large incisional wounds after abdominal surgery, rather than covering them with gauze (B, 2b) (2) Although negative-pressure wound therapy at primary closure during abdominal surgery may reduce incisional SSI, the indications and costs need to be considered (B, 3)

To increase dissemination of the guidelines, they were also published as a small booklet, and were uploaded onto the homepage of the Japan Society for Surgical Infection and Medical Information Network Distribution Service (Minds). This is a summary of the Guidelines for the prevention, detection, and management of gastroenterological SSI published in Japanese by the Japan Society for Surgical Infection in 2018. References and a funnel plot of meta-analysis are provided in a Supplemental File.

## Conflict of Interest and revision of the guidelines

The guidelines were supported only by the Japan Surgical Infection Society. The members of the Committee for Gastroenterological Surgical Site Infection Guidelines declared this conflict of interest to the society. Members who had a conflict with the CQs did not participate in casting their vote on the recommendation for the CQs. The guidelines will be revised in approximately 5 years.

## Preoperative management


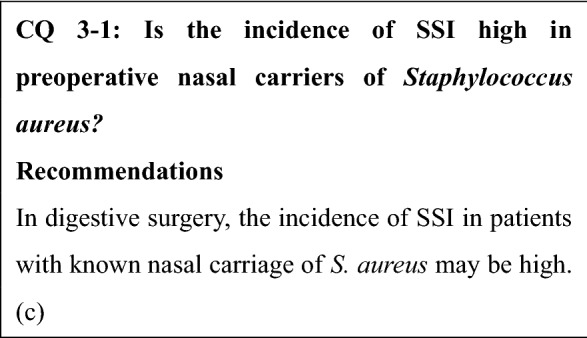


### Rationale

The meta-analysis was conducted on seven observational studies^(5–11)^. The incidence of *S. aureus* in SSI was significantly higher in nasal carriers of *S. aureus* undergoing digestive surgery (odds ratio [OR] 9.0, 95% confidence interval [CI] 5.09–15.91) (Fig. 3-1). However, six of the seven studies included patients who were undergoing not only digestive surgery, but also other types of surgery^(5, 7–11)^. Moreover, six of seven subjects were evaluated for the presence or absence of MRSA carriage only^(5–10)^.
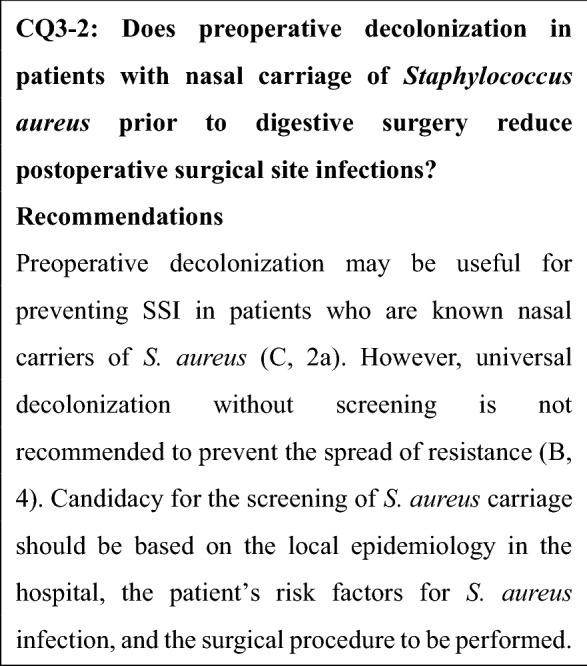


### Rationale

Two meta-analyses were performed to evaluate the benefit of decolonization in patients with known nasal carriage of *S. aureus* and of universal decolonization in all preoperative patients. Two RCTs showed that decolonization in patients with nasal *S. aureus* carriage had significantly greater benefit than no treatment in reducing the *S. aureus* SSI rate (Fig. 3-2)^(7, 12)^. There was no significant difference in mortality (Fig. 3-3). A prospective intervention cohort study also showed that the SSI rate in the decolonized group was not significantly higher than that of non-carriers (OR 0.8, 95% CI 0.19–3.44)^(13)^. All subjects in the decolonization group had received preoperative intranasal mupirocin ointment with or without a combination of chlorhexidine gluconate body wash. Two subsequent RCTs showed that universal decolonization did not reduce the SSI incidence significantly (Fig. 3-4)^(7, 14)^. On the other hand, in a historical control study the incidence of SSI was significantly lower with universal decolonization than without it^(15)^. However, the spread of mupirocin resistance by universal decolonization is an important issue. For this reason, the committee members decided not to recommend universal decolonization (B, 4).
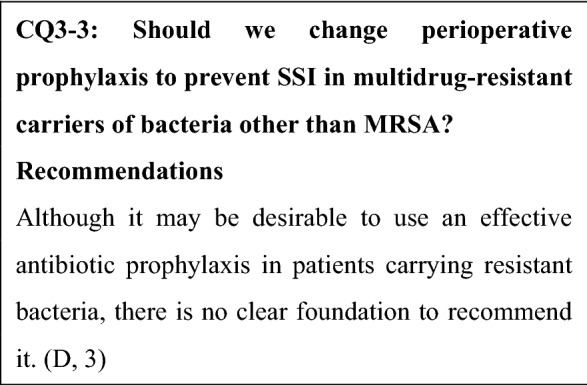



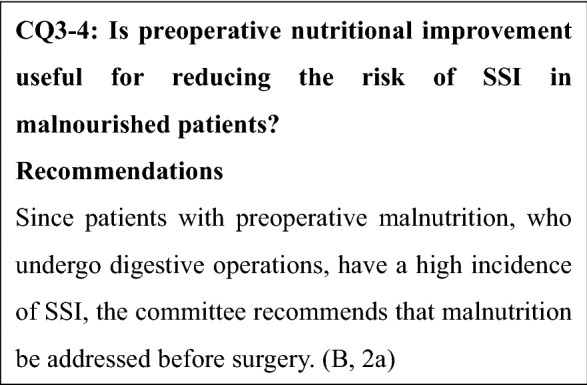


### Rationale

An analysis of six observational studies showed that the SSI rate in patients with preoperative malnutrition was higher than that in patients without malnutrition (OR 3.48, 95% CI 2.57–4.71, P < 0.00001) (Fig. 3-5)^(16–20)^. The reports used the prognostic nutrition indicators of serum albumin levels, prealbumin levels, and weight loss, to identify patients with malnutrition. A subsequent meta-analysis, consisting of two RCTs^(21, 22)^ and one observational study^(17)^ for SSI, revealed that nutrition improvement before surgery for patients with malnutrition reduced the incidence of SSI. The combined OR was 0.56 (95% CI 0.37–0.84) for the RCTs (Fig. 3-6) and 0.25 (95% CI 0.11–0.56) for the observational study. However, the methods and duration of appropriate nutrition interventions are unclear.
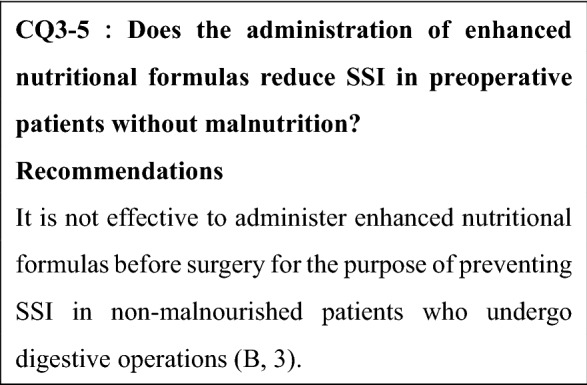


### Rationale

Three RCTs and one prospective assignment study showed that preoperative immunonutrition does not reduce the rate of SSI significantly in patients without malnutrition (RR 0.63, 95% CI 0.31–1.27) (Fig. 3-7). Moreover, preoperative immunonutrition did not result in significant improvement in terms of duration of hospital stay (Fig. 3-8)^(23, 24)^ [1, 4] and survival rates (Fig. 3-9)^(22, 23, 25)^. However, several studies have added immunomodulatory nutrition to regular meals, so the study design may not have been appropriate.
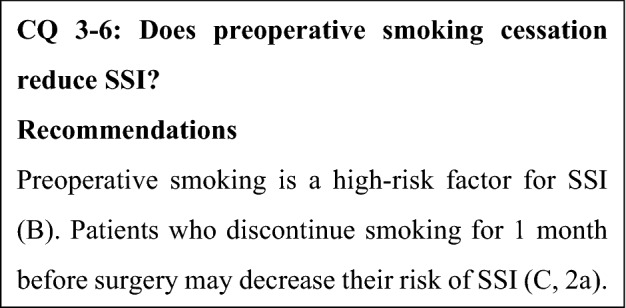


### Rationale

An RCT^(26)^ comparing SSI incidence in smokers with or without preoperative smoking cessation showed a tendency toward a decrease in the risk ratio (RR) of 0.48 (95% CI 0.2–1.16), but no significant difference was observed. In the meta-analysis comparing SSI incidence rates between preoperative smokers and nonsmokers, preoperative smoking was significantly associated with an SSI risk, with an OR of 1.79 (95% CI 1.59–1.94)^(27–57)^.
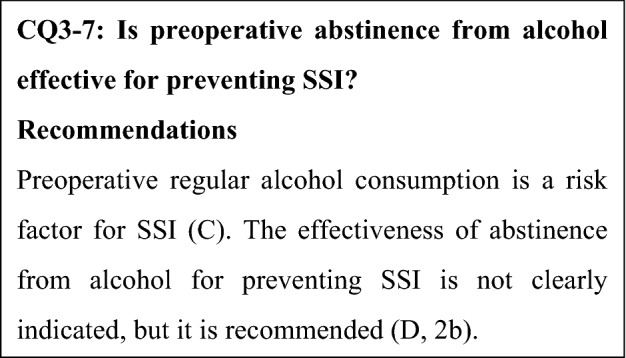


### Rationale

With respect to the incidence of SSI in relation to differences in alcohol consumption, the OR was 1.43 (95% CI 1.10–1.85) in seven observational studies (Fig. 3-11), and heavy drinking was a significant risk factor for SSI^(39, 58–63)^. A small RCT of preoperative abstinence in drinkers did not show a significant effect on SSI reduction (RR 0.972; 95% CI 0.70–1.35)^(64)^.
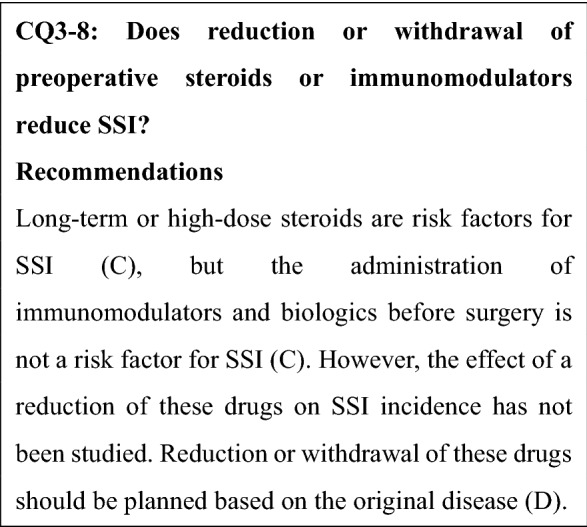


### Rationale

The administration of infliximab (IFX) has not been identified as a risk factor for SSI in patients with inflammatory bowel disease (OR 0.94; 95% CI 0.62–1.41)^(65–78)^. In an observational study by Ahn et al., the onset of postoperative infection, with or without long-term steroid administration, was examined in patients with inflammatory bowel disease. The steroid administration group had a significantly higher incidence of postoperative infectious complications (OR 5.83; 95% CI 1.063–32.021)^(79)^. In a report by Miki et al.^(80)^, postoperative infectious complications were examined by dividing the preoperative steroid total dose of 12 g as the boundary into two groups of high dose and low dose. The low-dose group had significantly lower postoperative infectious complications than the high-dose group (OR 3.40; 95% CI 1.172–9.862). Based on these results, long-term or high-dose steroids are regarded as a risk factor for postoperative SSI incidence (C).
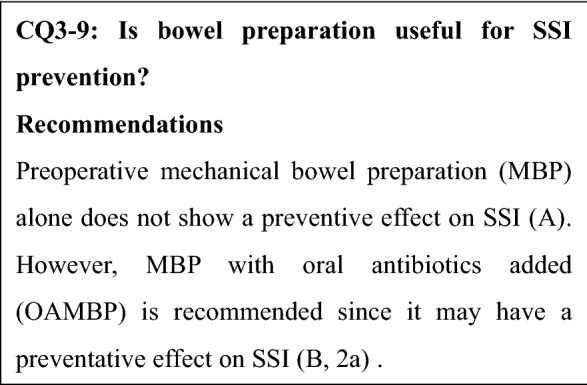


### Rationale

Ten RCTs that analyzed the presence or absence of MBP in colorectal surgery and the incidence of SSI^(81–90)^ found no difference between the RR of 1.02 (95% CI 0.82–1.28). In a comparison between OAMBP and MBP in colorectal surgery, there was a RR of 0.61 (95% CI 0.46–0.82) based on the analysis of the 10 RCTs^(901–100)^; OAMBP was found to reduce the rate of SSI significantly. Two large multiple-case studies compared OAMBP and no MBP in colorectal surgery.^(101, 102)^ The OR was 0.42 (95% CI 0.35–0.50), which was significant, and SSI reduction effect was also significantly higher for patients who received OAMBP.
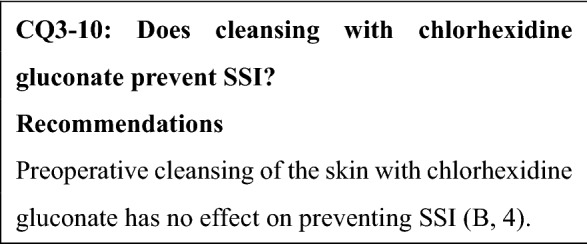


### Rationale

The meta-analysis^(103–112)^ showed that cleansing the skin with chlorhexidine did not reduce the occurrence of SSI (RR 0.94; 95% CI 0.85–1.05).
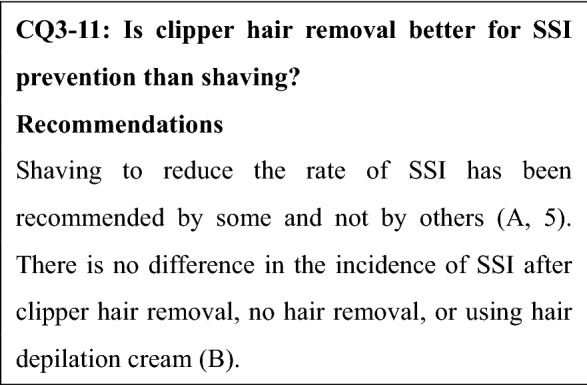


### Rationale

In a meta-analysis comparing clipper hair removal, depilation and shaving^(113–119)^, the RR of 0.54 (95% CI 0.38–0.78) was significant and the incidence of SSI was significantly lower after clipper hair removal. In the comparison of depilatory cream and shaving, the SSI was lower after the depilatory cream, not significantly^(120–124)^ (RR 0.52; 95% CI 0.24–1.11). In the comparison between no hair removal and shaving^(113, 121, 125–128)^, the RR was 0.58 (95% CI 0.34–0.98), which was significant, and the incidence of SSI was low in the absence of hair removal.

## Prophylactic antibiotics


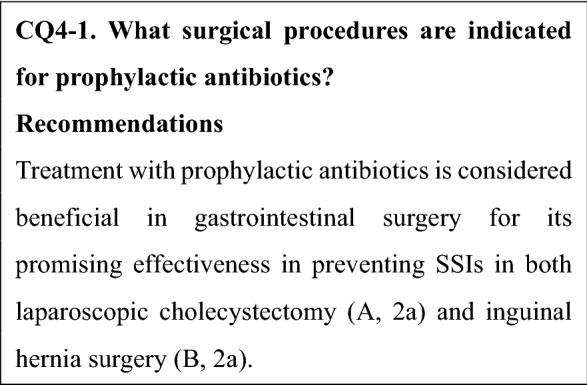


### Rationale

Figure 4-1 shows the results of a meta-analysis of 13 RCTs evaluating prophylactic antibiotic treatment for laparoscopic cholecystectomy^(129–141)^. Prophylactic antibiotic treatment resulted in a significant reduction in SSI incidence, and given that population sizes were sufficiently large, at 2000 patients in each RCT, the information was given a **level of evidence of A**. Similarly, Fig. 4-2 shows the results of a meta-analysis of the effects of prophylactic antibiotic treatment for inguinal hernia surgery. The results of these analyses demonstrate that prophylactic antibiotic treatment is effective, even for laparoscopic cholecystectomy and radical inguinal hernia surgery, both of which involve a low degree of surgical site contamination. Therefore, one can infer that prophylactic antibiotic treatment is effective in the field of gastrointestinal surgery with a higher degree of contamination and incidence of SSIs. Hence, prophylactic antibiotic treatment is deemed beneficial in gastrointestinal surgery.
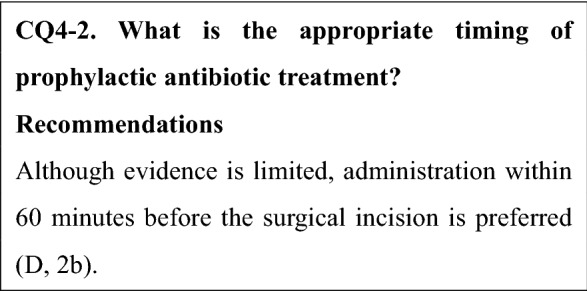


### Rationale

Three cohort studies compared SSI incidence after administration within 1 h before the surgical incision versus more than 1 h before the surgical incision^(142–144)^. Overall, 3606 patients received prophylactic antibiotic treatment within 1 h before the surgical incision and 3386 received prophylactic antibiotic treatment more than 1 h before the surgical incision. Analysis revealed an OR of 0.91 (95% CI 0.71–1.15) and no significant difference between the groups. However, theoretically, attaining an appropriate blood concentration of prophylactic antibiotics by the time of surgical incision is preferred, and hence the conventional administration method is recommended.
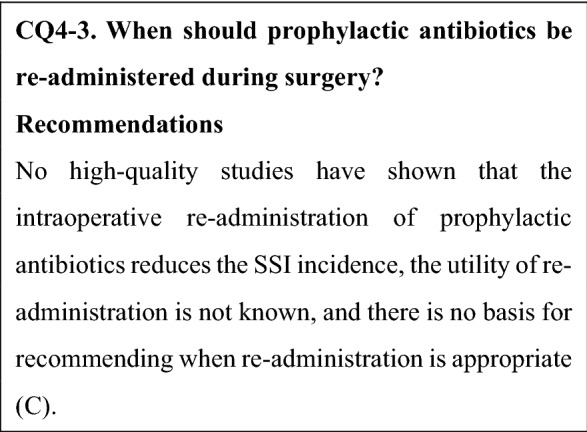


### Rationale

Although no high-quality study has shown that the SSI incidence is reduced by intraoperative re-administration of prophylactic antibiotics, from the viewpoint of PK and PD, it is logical that an appropriate blood concentration of antibiotics should be maintained during surgery, so intraoperative re-administration is preferred. Nevertheless, the appropriate timing of re-administration and how to adjust timing when severe bleeding occurs have not been established.
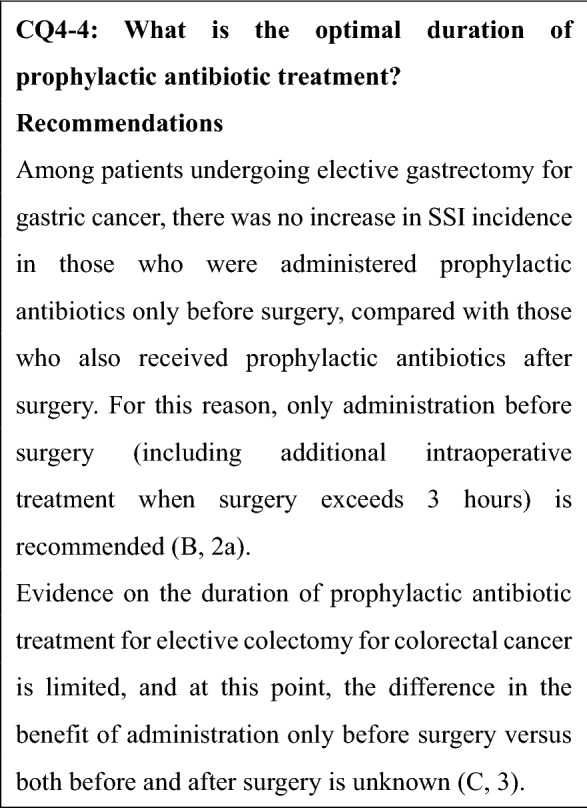


### Rationale

Figures 4-3 and 4-4 show forest plots of the SSI incidence after single dosing and repeated dosing of prophylactic antibiotics for gastrectomy and colectomy. Overall, the analysis for gastrectomy revealed a risk ratio (RR) of 0.97 (95% CI 0.55–1.68) and showed that SSI incidence was not increased significantly, even in the preoperative/intraoperative treatment group^(144–147)^. However, since the population size was not sufficiently large, this was given a **level of evidence of B**. Similarly, even in cases of elective colectomy for colorectal cancer, the SSI incidence did not increase significantly with single-dose treatment versus repeated-dose treatment. However, because there were only two RCT reports, the population size was small, and because the control group and intervention group differed greatly between the reports, the information was given a **level of evidence of C**.

## Intraoperative management


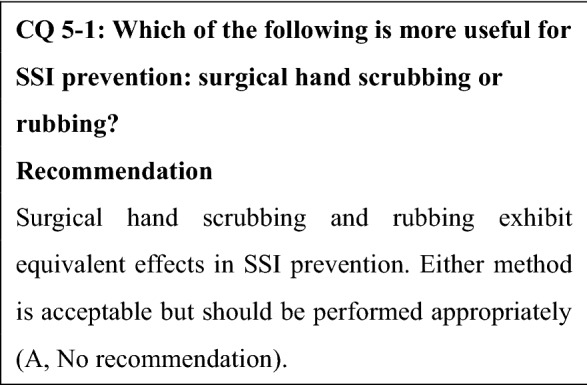


### Remarks

A similar description was found in some American^(148)^ and European^(149, 150)^ guidelines and no significant difference was found. There were three interventional RCTs with SSI incidence as the outcome, with the same conclusion^(151–153)^. Three observational studies with SSI incidence as outcome and three RCTs with a colony-forming unit (CFU) as an outcome were also confirmed^(151, 154–158)^. The superiority of either hygiene method has not been confirmed in any meta-analysis.

### Rationale

Rubbing with alcohol-based hand antiseptic is often performed instead of the conventional hand washing (scrubbing) with running water and soap for surgical hand hygiene. These methods have been compared in some guidelines^(148–150)^, but it has not been confirmed which method is superior. Intervention studies targeting SSI incidence as an outcome have not shown any superiority of either method. Moreover, observational studies using SSI incidence and RCTs with CFU as an outcome have not confirmed the superiority of either method.

A study from Japan identified some reports that the cost of the rubbing method is cheaper than the scrub method^(159)^, but the number of cases was low and the evidence level is low.
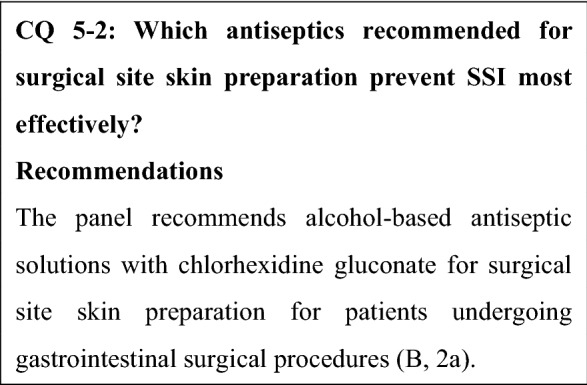


### Rationale

The most widely used agents for surgical skin preparation were povidone iodine or alcohol-based chlorhexidine gluconate solutions. The World Health Organization’s (WHO) 2016 Guidelines for the Prevention of Surgical Site Infections include recommendations for surgical site skin preparation^(150)^. Alcohol-based antiseptic solutions based on chlorhexidine gluconate for surgical site skin preparation were recommended. It is important to identify which skin antiseptics for surgical site skin preparation are most effective for preventing SSI.

The meta-analysis was conducted with three RCTs^(160–162)^. These three trials compared the efficacy of aqueous-based povidone iodine with alcohol-based chlorhexidine gluconate solutions for preventing SSI. The meta-analysis indicated that alcohol-based antiseptic solutions based on chlorhexidine gluconate are more effective than aqueous-based povidone iodine in reducing the risk of SSI in patients undergoing gastrointestinal surgery (Fig. 5-1). However, several factors require attention when applying this recommendation in clinical practice. The concentration of antiseptic agent varied among the studies, with the iodophor compound ranging from 5–10% and chlorhexidine gluconate ranging from 0.5–2.5%. Furthermore, we cannot use chlorhexidine gluconate solutions with a concentration over 1% in Japan. In future studies, we need to examine the appropriate concentration of chlorhexidine gluconate solutions for clinical practice in Japan. There are eight reports of a fire in the operating room associated with the use of alcohol-based antiseptics solutions in Japan. Measures should be taken to prevent such incidents before the use of alcohol-based antiseptic solution for surgical site skin preparation. We also need to check if patients have allergies to components of the antiseptics. Aqueous-based chlorhexidine gluconate should be used when a patient has alcohol intolerance.

Olanexidine has been introduced for surgical site skin preparation. Olanexidine has antimicrobial activity against a wide range of Gram-positive and Gram-negative bacteria. It also has bactericidal activity against *Vancomycin-resistant enterococci*, *Pseudomonas aeruginosa*, *Serratia *spp., and *Cepacia *spp. A clinical trial to elucidate whether these various concentrations antiseptics and Olanexidine are more effective than alcohol-based antiseptics should be performed.
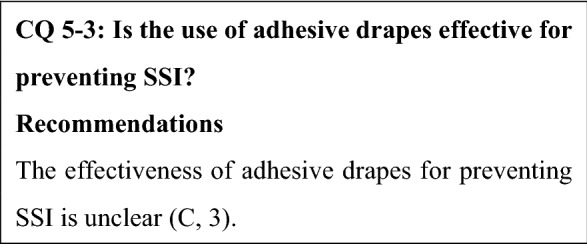


### Rationale

SSIs often develop after gastrointestinal surgical procedures; these wounds are mostly classified as clean-contaminated wounds. Various strategies, such as single-use drapes, are used to reduce wound contamination during a surgical procedure; however, their effectiveness in preventing wound infection is limited. For this reason, adhesive drapes are now used widely to reduce wound contamination.

We identified three RCTs^(164–166)^ and one observational study^(167)^. The observational study found that SSI was significantly less likely when surgery was performed with adhesive drapes. The meta-analysis was conducted with three RCTs^(164–166)^. Results from the meta-analysis indicated that surgery with adhesive drapes did not reduce the risk of SSI compared with surgery without adhesive drape (Fig. 5-2).
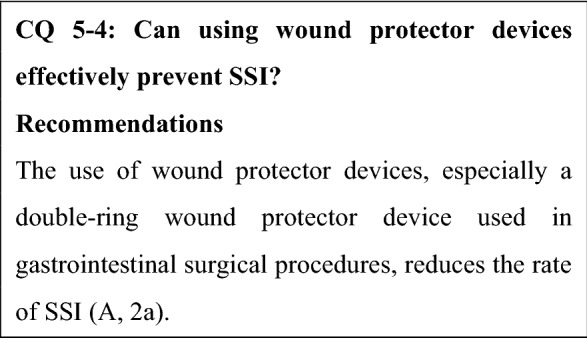


### Rationale

Despite using various strategies to minimize surgical wound contamination during surgery, incisional SSIs often occur after gastrointestinal surgical procedures. These wounds are usually classified as clean-contaminated wounds. Currently, surgical wound protectors, which comprise a non-adhesive plastic sheath attached to a single or double rubber ring that secures the sheath to the wound edges, are available to reduce wound edge contamination.

A meta-analysis was conducted with eight RCTs^(168–175)^ comparing the use of a surgical wound protector with conventional wound protection for preventing SSI. The meta-analysis indicated that wound protector devices are significantly more effective than conventional wound protection for reducing the risk of SSI in patients undergoing gastrointestinal surgery (Fig. 5-3). One trial performed an economic evaluation of wound protector devices^(176)^ and found no evidence to suggest that wound-edge protection devices are a cost-effective device to reduce SSI. There were several limitations in this analysis. First, the structural design of wound protector devices varied among the studies. Second, this analysis was unable to evaluate the efficacy in preventing SSI between the single-ring and the double-ring wound protector devices. Third, all studies targeted open gastrointestinal surgery. It is unknown whether the use of wound protector devices in laparoscopic surgeries can reduce the risk of SSI.
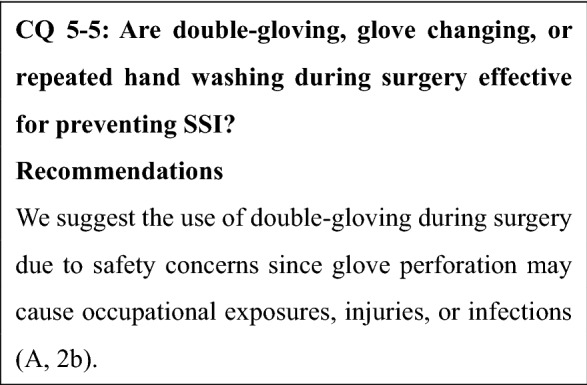


### Remarks

We cannot provide any recommendations for the use of double-gloving (D), changing gloves (C), or repeated hand washing (D) during digestive surgery for preventing SSI due to the lack of supporting evidence (D). However, double-gloving can reduce the exposure risk since the incidence of glove perforation is significantly lower for the inner gloves (A).

### Rationale

The WHO stated that a recommendation could not be formulated because of the lack of evidence^(150)^. However, it stated that most surgeons prefer to double-glove because bacterial contamination of the surgical field may occur in the event of glove perforation and most surgeons prefer to wear double gloves for their own protection against injury or infections.

There were no RCTs or observational studies concerning double-gloving for digestive surgery and the incidence of SSI. On the other hand, five RCTs were identified concerning glove perforations^(177–181)^. In the meta-analysis of these five RCTs, the incidence of perforation in single-gloving was significantly higher than that in the inner gloves used in double-gloving (Fig. 5-5). We could not find any RCTs in digestive surgery concerning changing gloves during surgery. Although we found an observational study with low quality evidence for changing gloves, there were no significant effects^(182)^. Moreover, we could not find any RCTs or OBSs in digestive surgery concerning repeated hand washing for preventing SSI. Thus, we could not formulate any recommendations for using double-gloving, changing gloves, or repeated hand washing during digestive surgery to prevent SSI. However, the incidence of glove perforation was significantly lower with double-gloving than with single-gloving.

The limitations of this analysis were as follows: (1) There was no cost analysis. (2) There was no evidence for whether glove perforation can actually lead to occupational infections. (3) Using double-gloving may reduce operability, although no evidence could be provided.
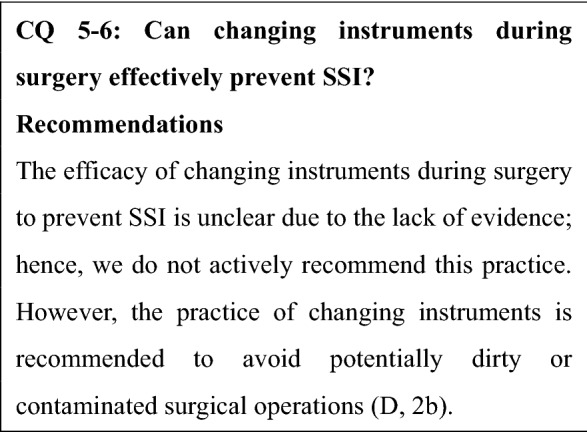


### Rationale

We could not address this CQ fully as there are no RCTs on the correlation between changing instruments and preventing SSI. Further studies are needed.
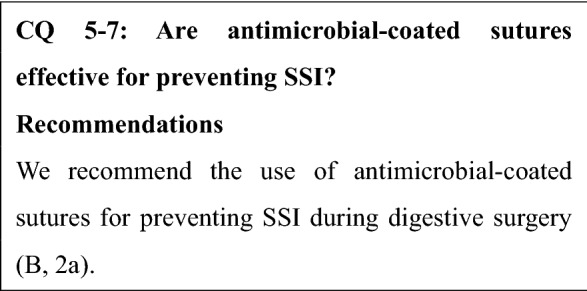


### Rationale

A total of 10 RCTs and 5 observational studies were identified^(183−197)^. We analyzed the incidence of SSI and the duration of hospitalization.

The results of a meta-analysis of all 10 RCTs showed that antimicrobial-coated sutures used in digestive surgery were effective for preventing SSI (RR 0.68; 95% CI 0.48–0.95; p = 0.03) (Fig. 5-5). However, in the analyses distinct from suture material, we could find significant efficacy only for poly-filament suture (RR 0.45; 95% CI 0.26–0.77; p = 0.004) in six RCTs, but not even for these in four RCTs (RR 0.79; 95% CI 0.54–1.17; p = 0.24). Analysis of five RCTs showed that the duration of hospitalization did not decrease significantly when antimicrobial-coated sutures were used (risk differences [RD] − 0.5; 95% CI − 16.68–6.69; p = 0.40). Thus, we recommend the use of antimicrobial-coated sutures to prevent SSI in digestive surgery. However, studies on cost benefit, efficacy in a pediatric population, or adverse events are lacking. All analyzed results of this systematic review can be found in the report by Uchino et al.^(198)^.
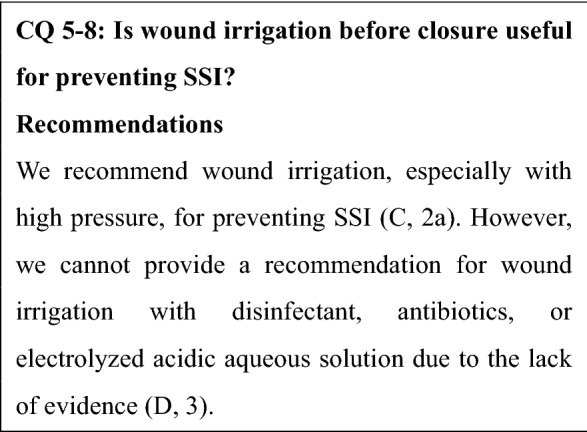


### Rationale

We could find only one RCT published since 2000, that examines the efficacy of wound irrigation in appendectomies^(199)^. In a systematic review and meta-analysis reported in 2015, wound irrigation was found to be significantly more effective in preventing SSI^(200)^. However, this meta-analysis included 15 appendectomies among 34 RCTs and 33 of the 34 studies were conducted before 2000. It is difficult to apply these results to recent cases of abdominal surgery, or even laparoscopic surgery. Therefore, recent evidence is insufficient on the relationship between wound irrigation and SSI.

We found two RCTs on high-pressure wound irrigation^(199, 201)^ in appendectomy and hepatic surgery, and three observational studies^(202−204)^. Although a significant effect was found in the meta-analysis for RCTs (Fig. 5-6) and OBSs (Fig. 5-7), evidence levels were low in both RCTs due to unclear definitions of outcome, randomization, concealment, and assessment of outcomes. Moreover, several methods of high-pressure irrigation were used in these studies, including pulsation and syringe with or without a thin needle.

Wound irrigation with disinfectant or antibiotic solution has not been practiced in digestive surgery since 2000.

There were two RCTs concerning the use of wound irrigation with electrolyzed acidic aqueous solution in preventing SSI^(205,206)^; however, no significant efficacy was observed for preventing SSI (RR 0.42; 95% CI 0.09–2.03; p = 0.19). Conversely, using electrolyzed acidic aqueous solution could be harmful since wound dehiscence and herniation were increased in one RCT (RR 2.28; 95% CI 1.03–5.04; p = 0.04)^(8)^. Based on these results, we recommend wound irrigation, especially with high pressure, for preventing SSI. However, a recommendation for wound irrigation with disinfectant, antibiotics, or electrolyzed acidic aqueous solution could not be formulated due to the lack of evidence in digestive surgery.
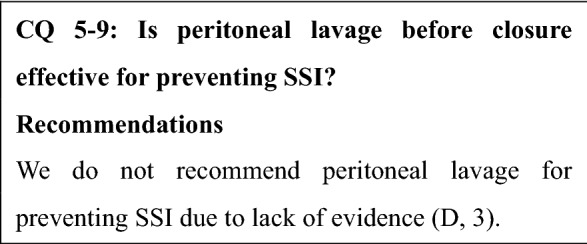


### Rationale

Figures 5-8 and 5-9 show the results of a meta-analysis of three RCTs^(207−209)^ and two observational studies^(210,211)^, respectively. Peritoneal lavage appeared to be harmful in one RCT on elective hepatic surgery, although there was no significant difference (RR 2.31; 95% CI 0.99–5.36)^(1)^. The other two RCTs were conducted on restricted to open appendectomy^(208,209)^. Two observational studies were also conducted on restricted to open appendectomy^(210,211)^. Although the RCTs did not find significant efficacy, analysis of the observational studies found that it was significantly harmful. However, we could not apply these results to universal digestive surgery for either emergency surgery with dirty/infected wounds or elective surgery with clean-contaminated wounds. Therefore, we cannot comment on the efficacy of peritoneal lavage for preventing SSI due to the lack of evidence.
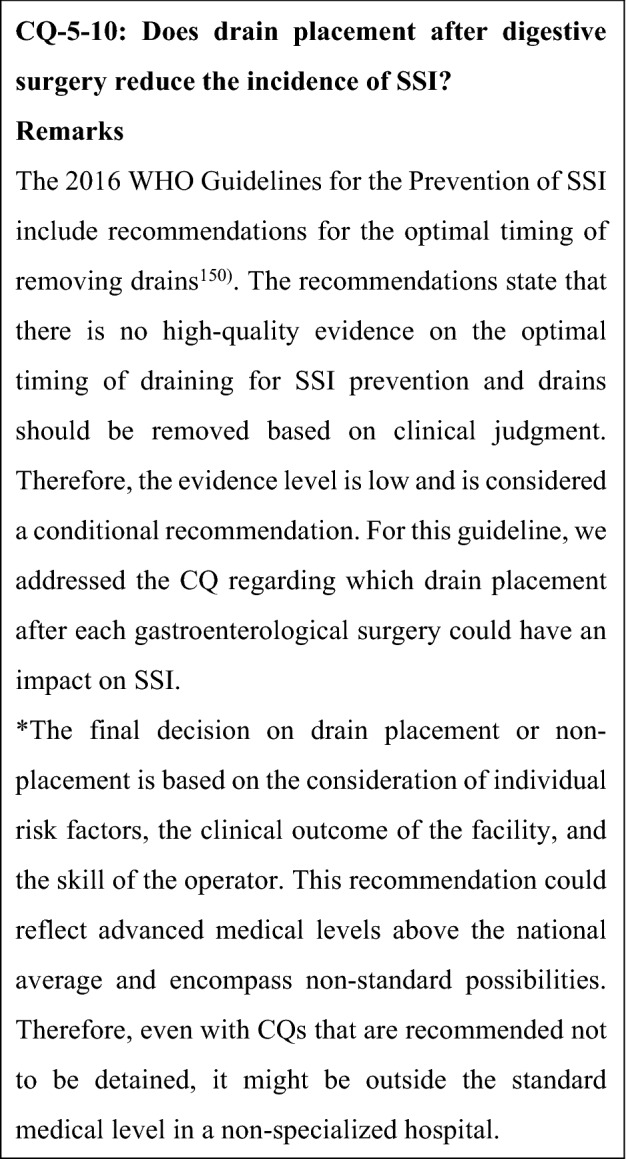

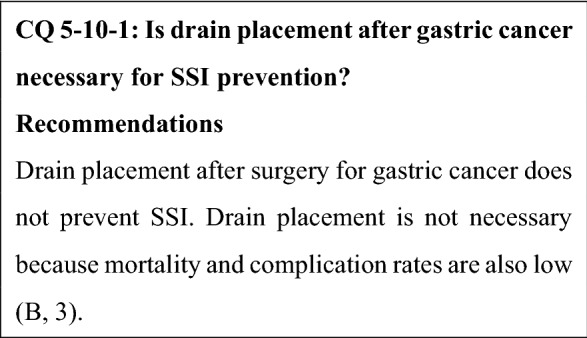


### Rationale

The reported RCTs^(212–214)^ were considered with few observational studies and high evidence. Two observational studies were also considered^(215, 216)^. There was no significant difference in mortality rates after total gastrectomy and distal gastrectomy with versus without drain placement (Figs. 5-10 and 5-11, respectively). We recommend that drain placement after gastrectomy be judged according to the clinical outcome and skills of the individual institution (B, 3).
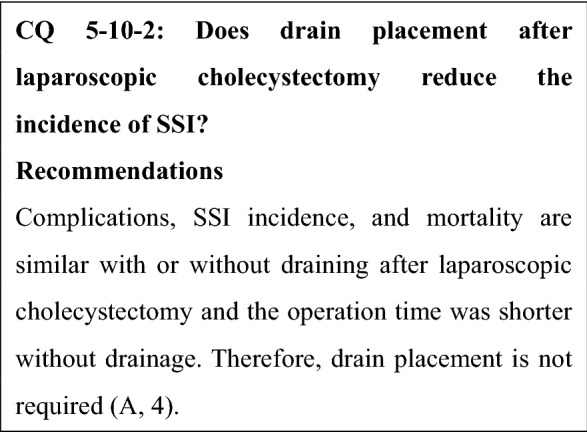


### Rationale

Results from a meta-analysis of 13 RCTs had a high evidence level because of the homogenous population and the inclusion of more than 500 cases^(217–229)^. There is no disadvantage for patients without drain placement because the mortality rate was not different with or without a drain. Moreover, the SSI rate did not differ among the groups, although that in the no-drain group tended to be lower than that in the drain group (Fig. 5-12). On the other hand, most investigators recommend no drain after laparoscopic cholecystectomy because of some benefits, such as shortening the operation time (Fig. 5-13). Therefore, committee members recommend that drainage after laparoscopic cholecystectomy is unnecessary (A, 4).
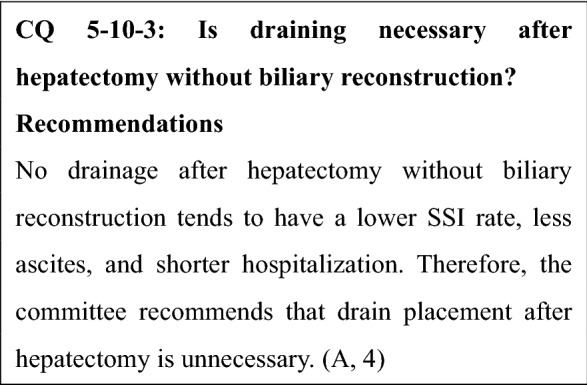


### Rationale

The meta-analysis was conducted with six RCTs^(230–235)^. Mortality rates were not significantly different between the drain and no-drain groups (Fig. 5-14). The SSI rates tended to be higher in the drain group (Fig. 5-15) and ascitic fluid leakage was significantly reduced in the no-drain group (Fig. 5-16). Therefore, as drain placement after hepatectomy without biliary reconstruction tends to increase SSI rates, ascitic leakage, extend the duration of hospitalization, no drain is recommended with high evidence levels (A, 4).
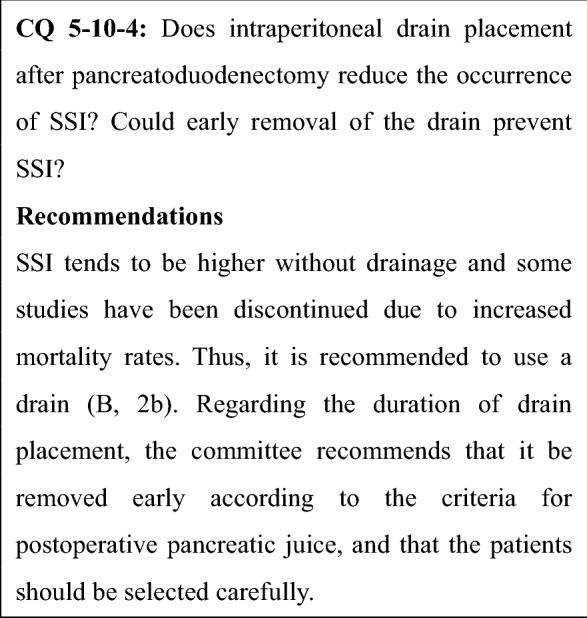


### Rationale

The meta-analysis was conducted with three RCTs^(236−238)^. The Van Buren’s (2014) RCT study^(237)^ for a pancreatoduodenectomy was discontinued by the study’s Medical Safety Commission (Fig. 5-17)^(239)^. Therefore, it is suggested that no drainage may increase the mortality rate, and drain placement is better from the viewpoint of medical safety (B, 2b). Concerning the timing of drain removal, the group that had early drain removal after surgery within certain removal criteria had fewer intraabdominal infections (Fig. 5-18), and the duration of hospitalization was also shortened significantly (Fig. 5-19). Early removal is recommended by the committee, although this does not have a high evidence level and the number of cases is limited.
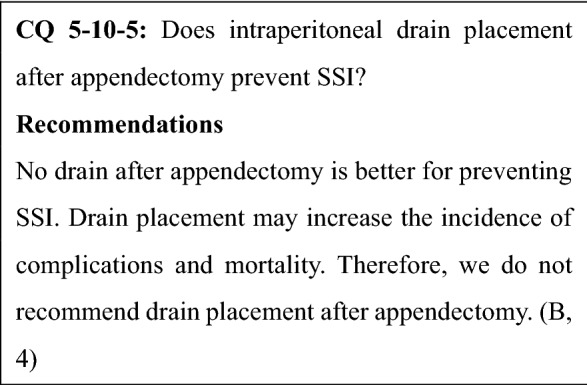


### Rationale

It is important to note that all evidence was collected for open surgery using a Penrose drain. Although the evidence level was moderate, most appendectomies are now performed under laparoscopy and the clinical background of the studies and medical reality differs. With this limited evidence, a meta-analysis was conducted with seven RCTs^(239−245)^. Mortality rates (Fig. 5-20) were lower in the no-drain group. Therefore, drain placement after appendectomy is unnecessary, unless patients need abdominal lavage.
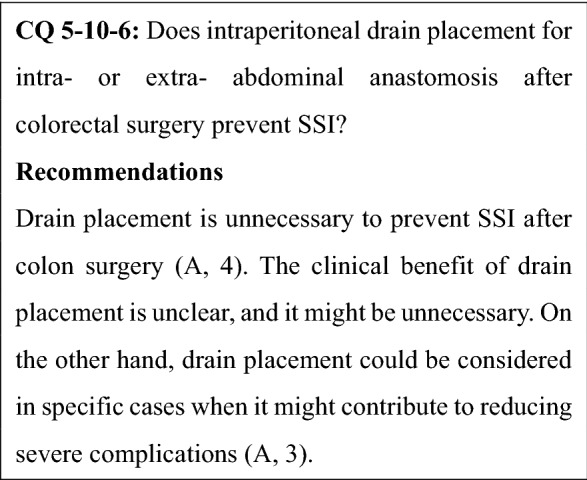


### Rationale

Drain placement does not affect the clinical outcome of suture failure, abscess formation, or mortality, after colon surgery or rectal surgery. Although there was no significant difference in rectal surgery, the incidence of mortality (Fig. 5-21) tended to be lower in the drain group^(246–249)^. In colon surgery, we did not identify any merits of drain placement or of no drain placement^(250–253)^. The committee recommend that drain placement is unnecessary (Recommendation 4). There was no significant difference in rectal surgery, and the merits of drain placement are unclear, although drain placement tends to reduce serious complications (Recommendation 3).
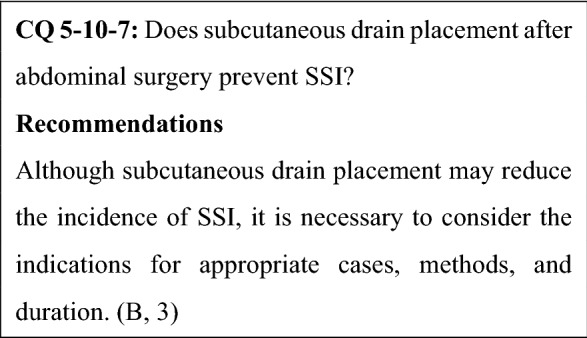


### Rationale

The meta-analysis was conducted with seven RCTs^(254–260)^, which showed that the incidence of superficial incision wounds in SSI was significantly reduced when subcutaneous drains were placed (Fig. 5-22). With respect to the high evidence level of the studies included in the meta-analysis, subcutaneous draining appears to reduce the incidence of SSI after gastrointestinal tract surgery (Evidence level, B). However, there is no clear recommendation because the subcutaneous drains in clinical practice are not widespread, and the indication in clinical practice is inconclusive.
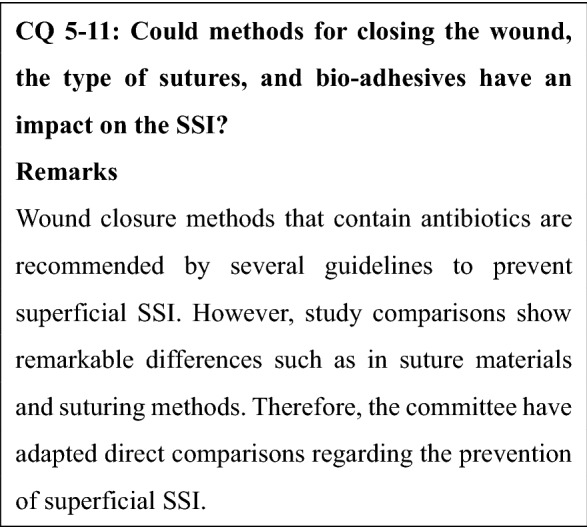



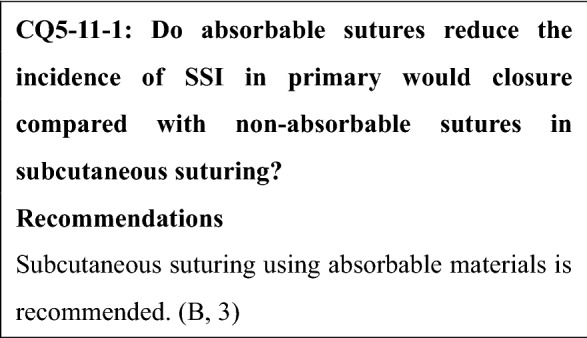


### Rationale

Meta-analysis of six RCTs for superficial SSI^(261−266)^ revealed a significantly lower incidence of SSI when absorbable sutures were used than when non-absorbable sutures were used (Fig. 5-23). The meta-analysis from appendectomy alone showed that the incidence of superficial SSI was significantly lower for absorbable sutures than non-absorbable sutures. Therefore, absorbable sutures are more clinically valuable than non-absorbable sutures for superficial SSI and wound dehiscence in primary closure using subcutaneous sutures^(261−266)^.
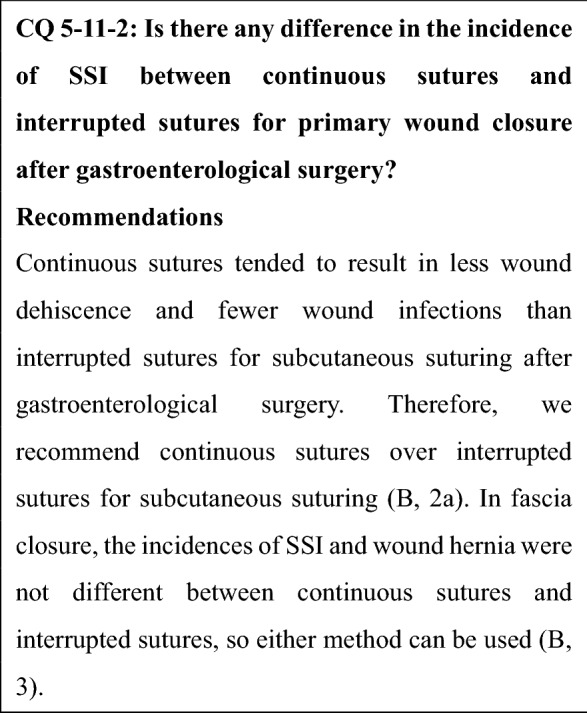


### Rationale

A meta-analysis of four RCTs for superficial SSI and wound dehiscence for subcutaneous suturing^(262, 263,267, 268)^ revealed that the incidence of wound dehiscence was significantly lower for continuous sutures than for interrupted sutures (Fig. 5-24). A meta-analysis of eight RCTs for superficial SSI and hernia formation for fascia closure^(269–276)^ revealed that the SSI rate and hernia formation were not different between continuous and interrupted sutures. Because there was less wound dehiscence associated with continuous sutures, interrupted sutures are strongly recommended for subcutaneous suturing.
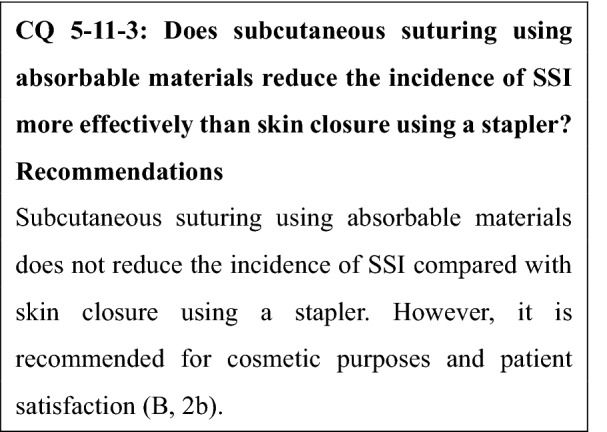


### Rationale

A meta-analysis of two RCTs on the rate of SSI rate and wound complications after skin closure using subcutaneous sutures versus a skin stapler^(277,278)^ revealed that the incidence of each clinical indicator for subcutaneous sutures tended to be less than that for skin stapling. The RCTs consisted of more than 1000 subjects and had high statistical power. Subcutaneous sutures resulted in less wound thickness than a skin stapler. Patient satisfaction after subcutaneous sutures were used ranked excellent and obtained 54% (268/511), whereas patient satisfaction after a skin stapler was used obtained only 42.7% (211/494: P = 0.002).
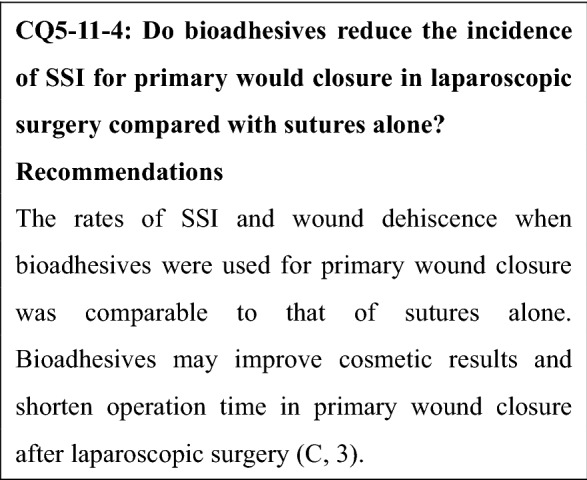


### Rationale

The number of patients in each study was small, so the evidence level was C. A meta-analysis of six RCTs that compared bioadhesives and subcutaneous sutures^(279–284)^ revealed that the incidence of SSI and wound dehiscence was similar for both methods. The cost of using bioadhesives could be higher than that of subcutaneous sutures and it can also cause chemical burns. Therefore, the clinical application of these materials must be carried out carefully.

## Perioperative management


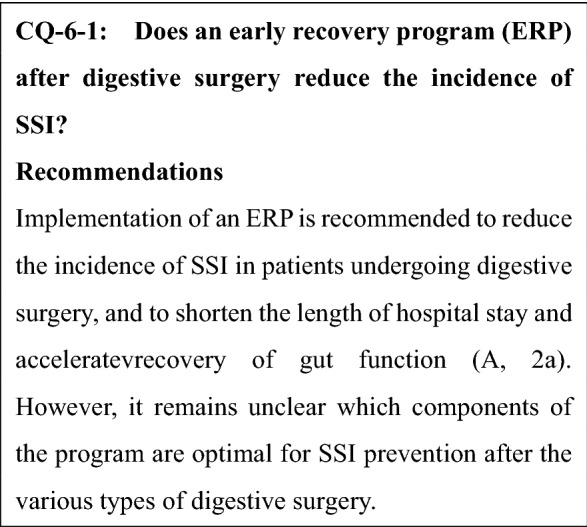


### Rationale

Figure 6-1 shows the results of a meta-analysis of 29 RCTs^(285−313)^ on early recovery after surgery (ERAS)/Fast Track Surgery (FTS) for digestive surgery. Implementation of an ERP like ERAS/FTS significantly reduced the incidence of SSI after digestive surgery. The length of hospital stay (standardized mean difference [SMD]: − 1.05 day; 95% CI − 1.41, − 0.75) and overall postoperative complications (RR 0.76; 95% CI 0.63, 0.93) were significantly lower in the ERAS/FTS groups. Another meta-analysis of 27 RCTs also demonstrated that ERAS/FTS for abdominal or pelvic surgery had a similar effect on SSI prevention (RR 0.77; 95% CI 0.58, 0.98)^(314)^ [30]. Therefore, we recommend ERP after digestive surgery to decrease the risk of SSI (A, 2a). However, it remains unclear which program components are optimal to prevent SSI after various types of digestive surgery because several components constituting ERP were different in every type of digestive surgery reported.
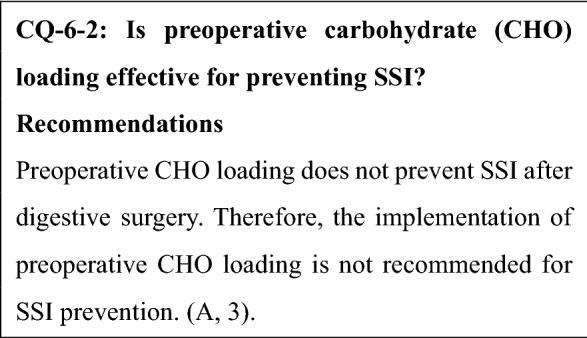


### Rationale

Meta-analysis of six RCTs^(315–320)^ on preoperative CHO loading did not show any effect on SSI prevention after digestive surgery (Fig. 6-2). Another meta-analysis of 8 RCTs ahowed no significant difference in the incidence of postoperative complications (RR 0.85; 95% CI 0.66, 1.08) between preoperative CHO loading and a placebo^(315–322)^. A meta-analysis of preoperative CHO loading for elective surgery, including cardiovascular and hip joint surgery, also showed no effect on preventing surgical complications (RR 0.88; 95% CI 0.50, 1.55)^(323)^. Therefore, we do not recommend the preoperative administration of CHO to prevent SSI (A, 3).
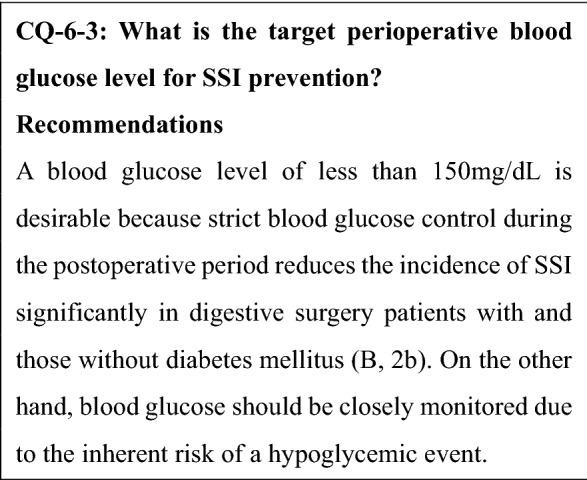


### Rationale

The American College of Surgeons (ACS)/Surgical Infection Society (SIS) guideline recommends a target blood glucose level to prevent SSI of 110–150 mg/dL, and less than 180 mg/dL for cardiovascular surgery^(324)^. The CDC guideline recommends a target blood glucose level of less than 200 mg/dL^(325)^ and the WHO global guideline suggests 110–150 mg/dL or less than 150 mg/dL without a definitive recommendation^(150)^. To address the optimal blood glucose target level for SSI prevention in digestive surgery, four RCTs^(326−329)^ and three observational studies^(330–332)^ were identified. In a meta-analysis of the four RCTs, the summary estimate showed a significant benefit of intensive glucose control compared with conventional control for reducing the incidence of SSI in patients with and those without diabetes (Fig. 6-3). The intensive group’s target blood glucose levels were 80–110 mg/dL in the four RCTs, resulting in a high incidence of hypoglycemic events (RR 7.11, 95% CI 2.15, 23.55). The target levels of blood glucose were different in each observational study: 80–140 mg/dL, less than 125 mg/dL, and less than 180 mg/dL, respectively. The incidences of SSI in each glucose control group were significantly lower than those in the reference groups. A recent meta-analysis reported that the effect was similar in studies with a target blood glucose level of less than 110 mg/dL (RR 0.50; 95% CI 0.35, 0.73) and an upper limit target level of 110–150 mg/dL (RR 0.43; 95% CI 0.29, 0.63)^(333)^. Considering this evidence, we recommend blood glucose target levels of less than 150 mg/dL to reduce the incidence of SSIs in digestive surgery patients with and those without diabetes mellitus; however, the available evidence is low quality and hypoglycemic events should be avoided in intensive glucose control (B, 2b).
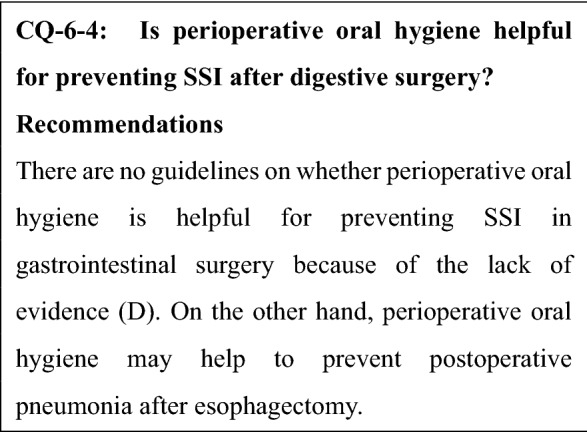


### Rationale

We did not identify any RCTs or observational studies investigating the relationship between oral hygiene and the incidence of SSI in digestive surgery in English language journals. Only one observational study found that preoperative dental care significantly reduced severe pneumonia after esophagectomy (Fig. 6-4)^(334)^. The evidence level of this retrospective study was low. However, preoperative oral care appears to have become adopted widely in the field of cancer surgery. We decided not to provide a recommendation about preoperative oral care and SSI prevention.
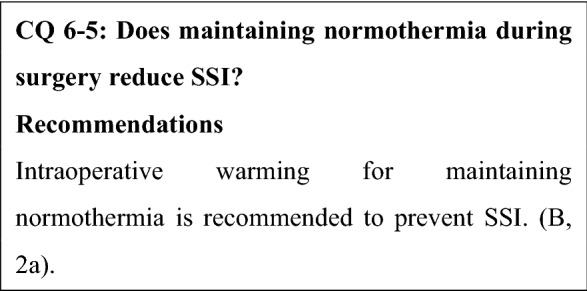


### Rationale

A meta-analysis was conducted with two RCTs^(335,336)^ (Fig. 6-5). Intraoperative warming and maintaining normothermia significantly reduces the risk of SSI after surgery compared with non-warming care. However, the evidence level is moderate (B) because the two RCTs contained small sample sizes and there have been no recent large-scale studies on digestive surgery. Intraoperative hypothermia causes not only SSI but also postoperative shivering, delayed emergence from anesthesia, and abnormal coagulation. Thus, patients should be warmed and normothermia with a core temperature > 36 °C maintained during surgery using methods such as forced-air warming, warming blankets, and a fluid warmer.
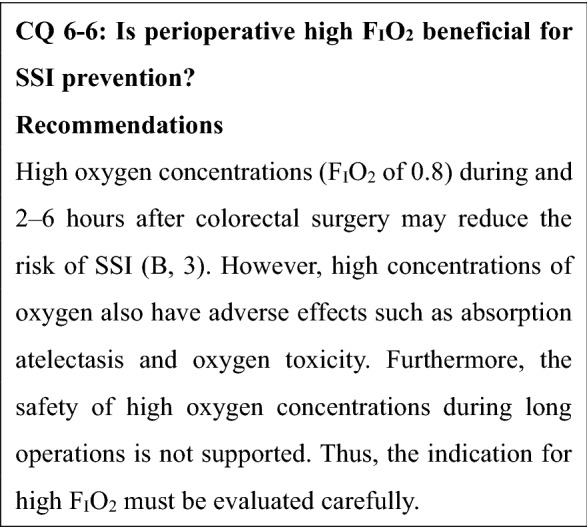


### Rationale

The meta-analysis was conducted on 10 RCTs on digestive surgery^(337−346)^. High perioperative oxygen concentrations (F_I_O_2_ of 0.8) do not reduce the risk of SSI significantly after digestive surgery, compared with the usual standard of care (F_I_O_2_ of 0.3–0.35) (Fig. 6-6). However, when meta-analysis was conducted on four RCTs on high-concentration oxygen administered during and 2 h or more after colorectal surgery^(337, 339,340,346)^, high F_I_O_2_ during and 2–6 h after surgery reduced the risk of SSI after colorectal surgery significantly (Fig. 6-7).

The indication for high perioperative F_I_O_2_ should be evaluated carefully, especially for patients with respiratory diseases such as chronic obstructive pulmonary disease and interstitial pneumonia for whom high oxygen concentrations may exacerbate respiratory failure. In the 10 RCTs on digestive surgery included in this meta-analysis, no harmful injury was reported in the control group, or after the administration of high-concentration oxygen for about 3 h during surgery and up to 6 h after surgery. On the other hand, the effect of high F_I_O_2_ during long operations has not been verified, and its safety is unknown. The beneficial effect of perioperative high F_I_O_2_ on SSI prevention is limited, and its safety is unclear. Therefore, the committee members do not recommend it (recommendation level 3). Further research is needed.
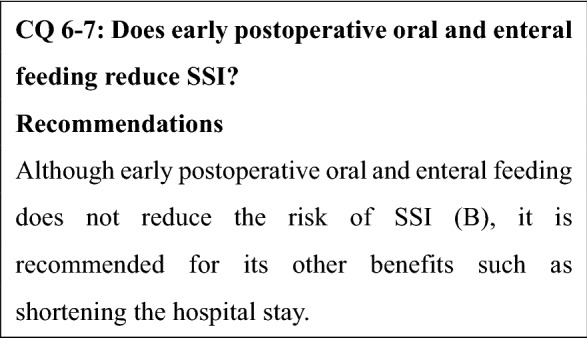


### Rationale

A meta-analysis was conducted with seven RCTs on digestive surgery (Fig. 6-8)^(347–353)^. Early postoperative oral and enteral feeding does not prevent SSI. The meta-analysis did not show that early postoperative oral and enteral feeding was useful for SSI prevention. However, it is an established element of the ERAS protocol and its usefulness for shortening the length of hospital stay and other advantages is well documented. Therefore, the committee members recommend it.

## Wound management


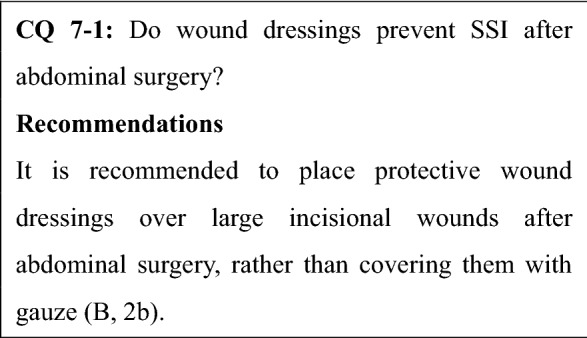


### Rationale

The results of a meta-analysis of eight RCT reports on the combination of hydrocolloid material and silver-containing wound protection material^(355−362)^ showed that the material for each control varied. Thus, it was considered evidence level B. In the meta-analysis, the use of any protective material reduced the incidence of wound infection significantly (Fig. 7-1). Despite detection bias or technique-related inconsistencies, wound infection is reduced by protective dressings (B, 2b).
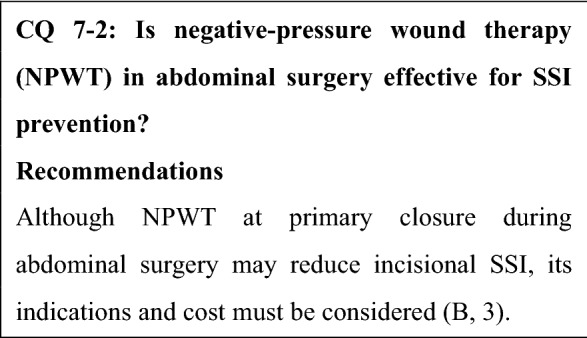


### Rationale

NPWT for primary incisional wounds had a significantly lower incidence of SSI than gauze dressing from the meta-analysis of four RCTs^(363−366)^ (Fig. 7-2). Seroma formation also tended to be lower with NPWT although the difference was not significant (Fig. 7-3). Thus, NPWT may reduce the incidence of SSI in primary incisional wounds. This recommendation has been made based on consideration that the indication of the disease and the type of surgical procedures varied. The negative pressure and the period were also unstable. Furthermore, Japanese public insurance does not yet cover NPWT for primary incisional wounds.

## Electronic supplementary material

Below is the link to the electronic supplementary material.

Supplementary file1. (DOCX 2281 kb)
