# Supplementary material for: The Japan Society for Surgical Infection: guidelines for the prevention, detection, and management of gastroenterological surgical site infection, 2018
Source: Surg Today. 2020 Dec 15;51(1):1–31. doi: 10.1007/s00595-020-02181-6 (PMC7788056; doi:10.1007/s00595-020-02181-6)

**Chapter 3. Preoperative management**

**Fig. 3-1. Rate of SSI according to the presence vs. absence of nasal *S. aureus***


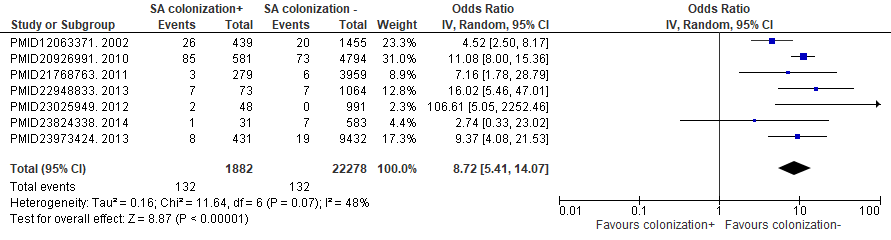


**Fig. 3-2****. Rate of SSI in nasal *S. aureus* carriers with vs. without decolonization**


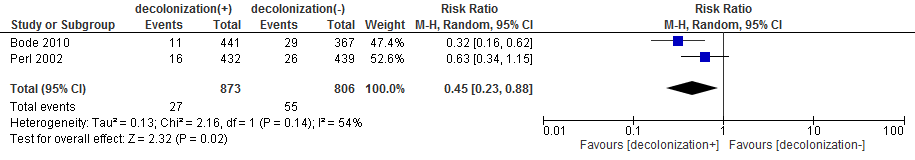


**Fig. 3-3. Mortality associated with SSI for nasal *S. aureus* carriers with vs. without decolonization**


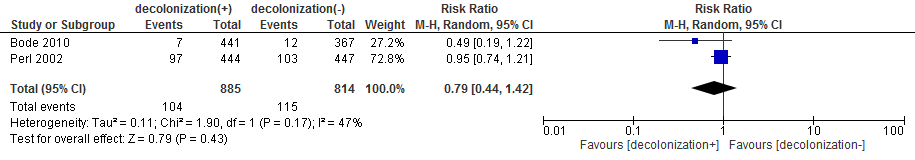


**Fig. 3-4. Rate of SSI according to universal decolonization vs. without universal decolonization**


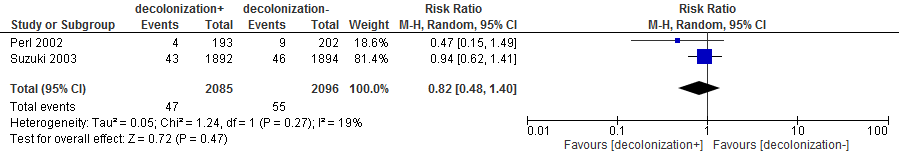


**Fig. 3-5. Rate of SSI in patients with malnutrition vs. in those without malnutrition**


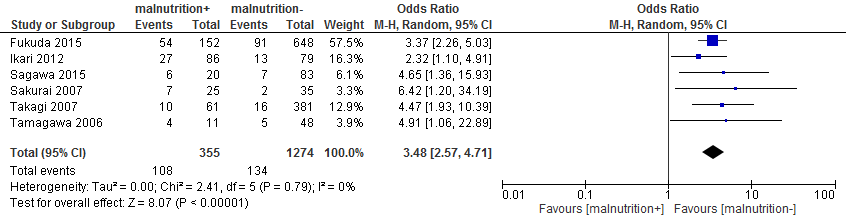


**Fig. 3-6. Rate of SSI in patients with malnutrition with vs. without preoperative nutritional improvement**


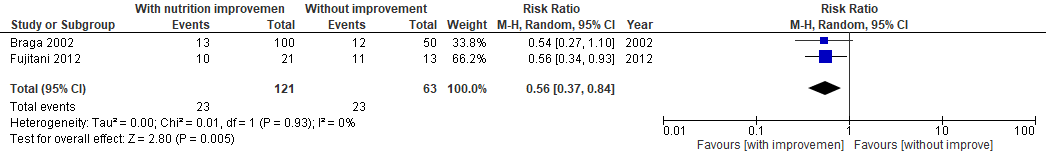


**Fig. 3-7. Rate of SSI in digestive surgery patients without malnutrition with vs. without preoperative enhanced nutritional formulas**


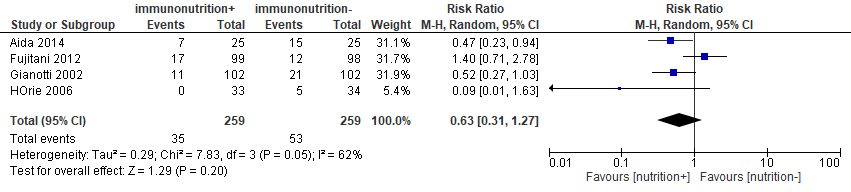


**Fig. 3-8. Duration of hospital stay (days) after digestive surgery for patients without malnutrition with vs. without preoperative enhanced nutritional formulas**


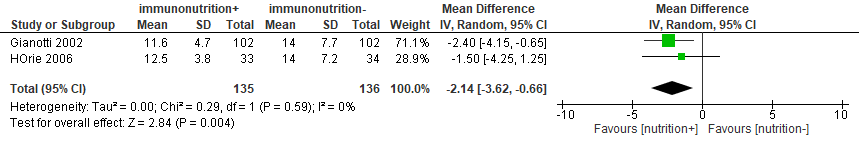


**Fig. 3-9. Survival rate of patients without malnutrition undergoing digestive surgery with vs. without preoperative enhanced nutritional formulas**


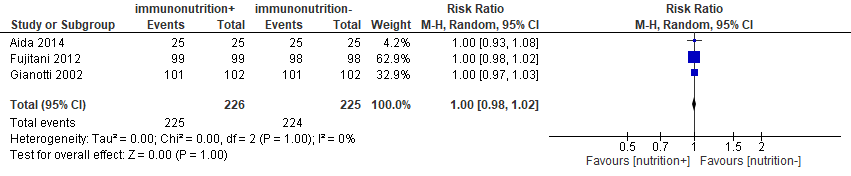


**Fig. 3-10. SSI incidence after gastrointestinal surgery in preoperative smokers and nonsmokers**


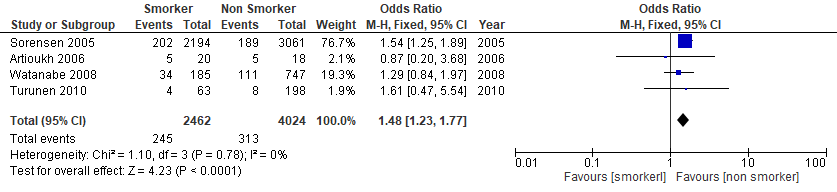


**Fig. 3-11. Meta-analysis of the impact of preoperative alcohol consumption on SSI incidence**
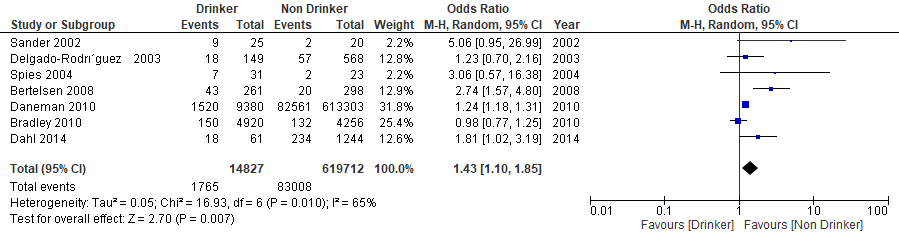


**Fig 3-12. SSI rate according to the administration of IFX to patients with inflammatory bowel disease**


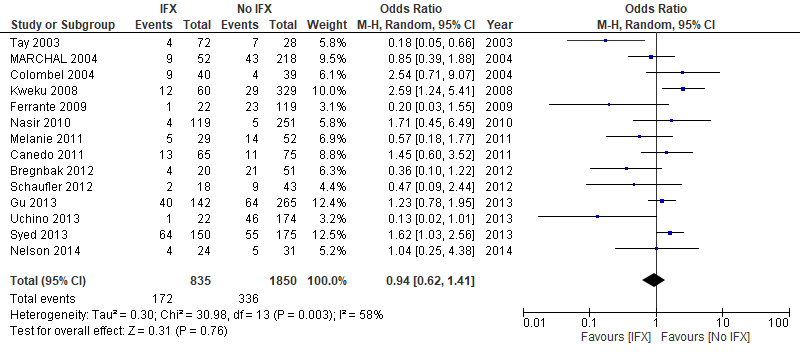


**Fig. 3-13. Incidence of SSI in patients administered MBP prior to colorectal surgery**


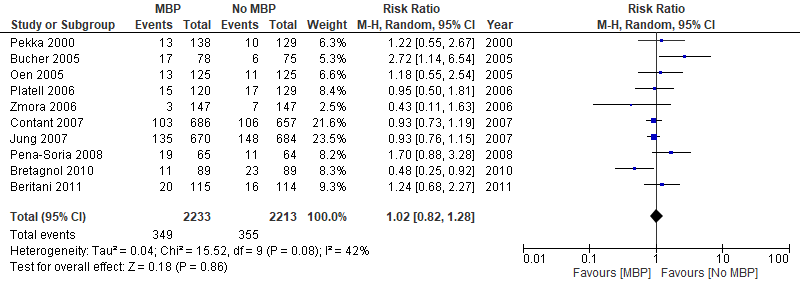


**Fig. 3-14. SSI incidence among patients with OAMBP or MBP prior to colorectal surgery**
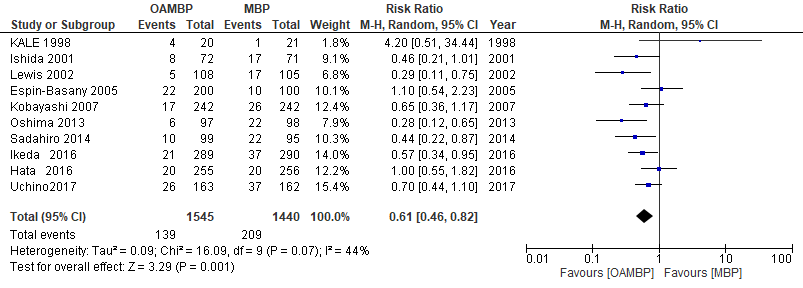


**Fig. 3-15. SSI incidence in patients with OAMBP but without MBP prior to colorectal surgery**
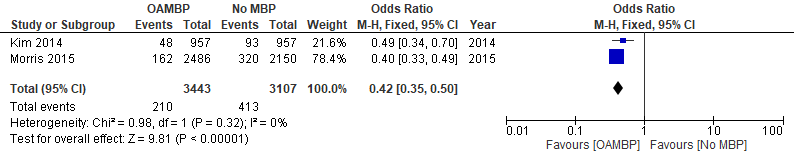


**Fig. 3-16. Incidence of SSI after preoperative cleansing with or without chlorhexidine gluconate**


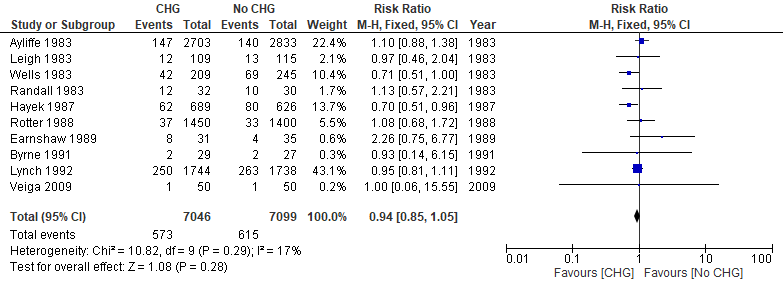


**Fig. 3-17. SSI incidence associated with hair removal by a clipper, depilation, or shaving**


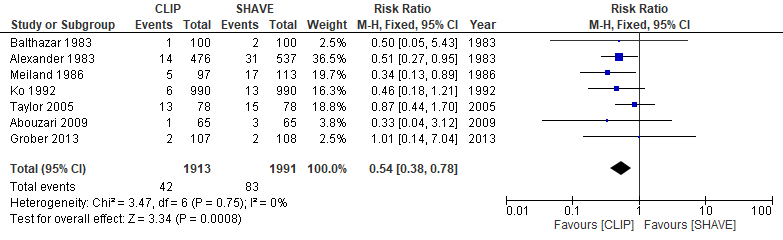


**Fig. 3-18. SSI incidence associated with depilatory cream or shaving**


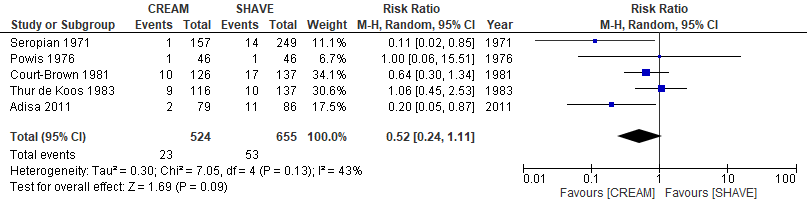


**Fig. 3-19. SSI incidence with no hair removal or shaving**


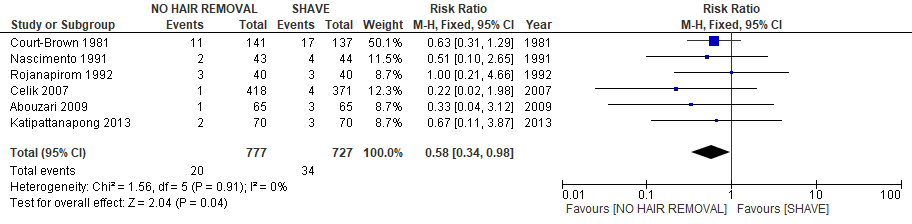


**Chapter 4. Prophylactic antibiotics**

**Fig. 4-1. Meta-analysis of prophylactic antibiotic treatment for laparoscopic cholecystectomy**


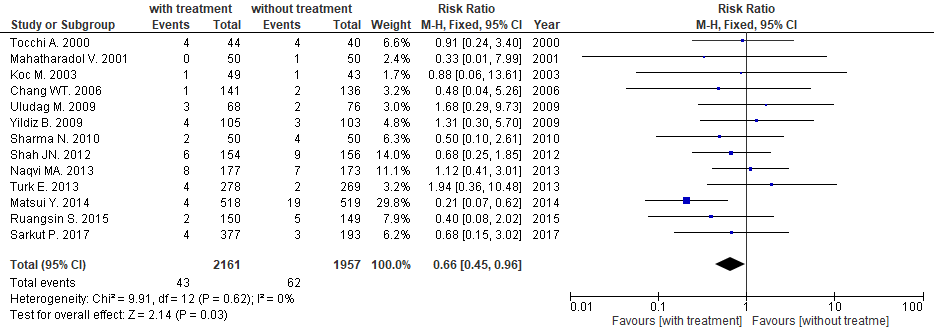


**Fig. 4-2. Meta-analysis of prophylactic antibiotic treatment for inguinal hernia surgery**


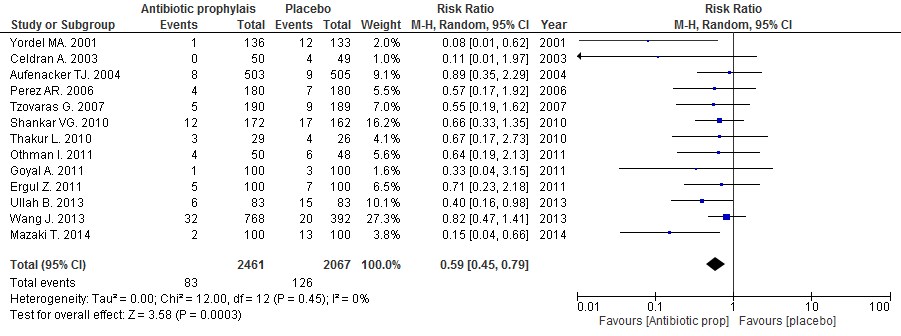


**Fig. 4-3. Meta-analysis of SSI incidence after single dosing vs. repeated dosing of prophylactic antibiotics for gastrectomy**


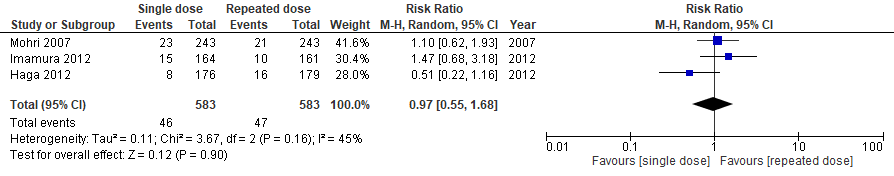


**Fig. 4-4. Meta-analysis of the duration of prophylactic antibiotic treatment for colectomy**


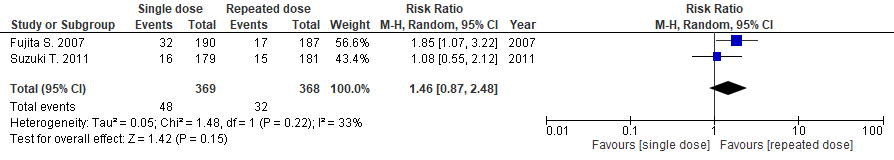


**Chapter 5. Intraoperative management**

**Fig. 5-1. SSI rate after chlorhexidine gluconate versus povidone iodine,**


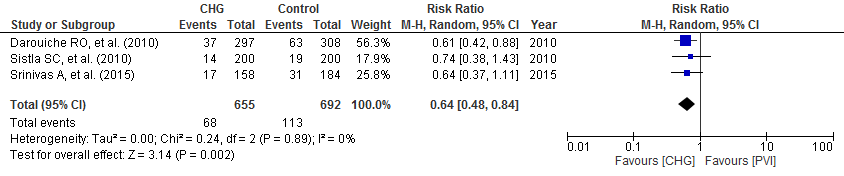


**Fig. 5-2. SSI rate with versus without the use of an adhesive drape**


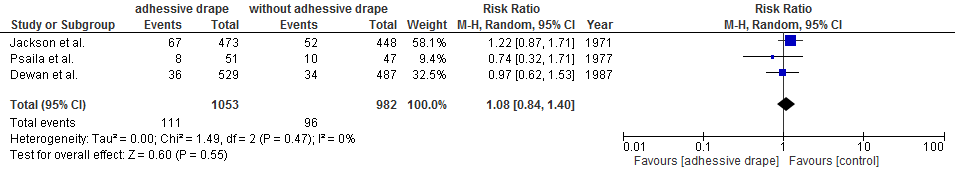


**Fig. 5-3. SSI rate after distal gastrectomy performed with aound protector device versus with conventional wound protection (control)**


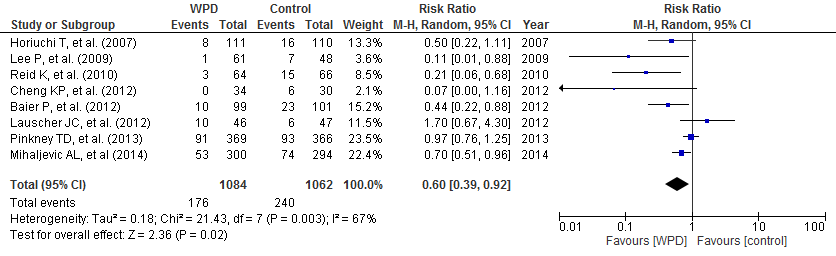


**Fig. 5-4. Forest plot of the meta-analysis for glove perforation (single vs. inner glove from double-gloving).**


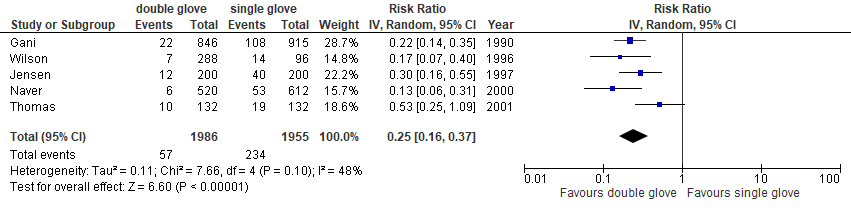


**Fig. 5-5. Forest plot of the meta-analysis of antimicrobial-coated sutures (RCTs).**


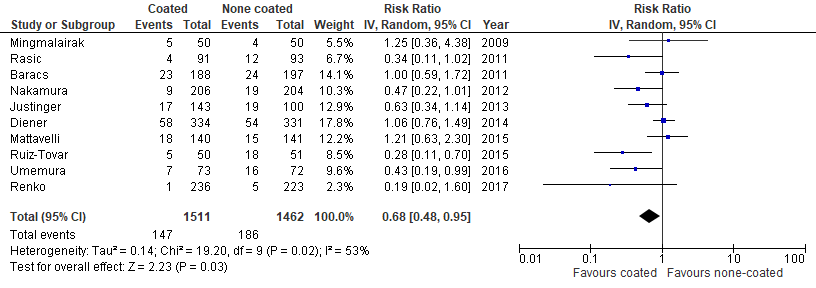


**ig 5-6: Forest plot of the meta-analysis of high-pressure wound irrigation (RCTs).**


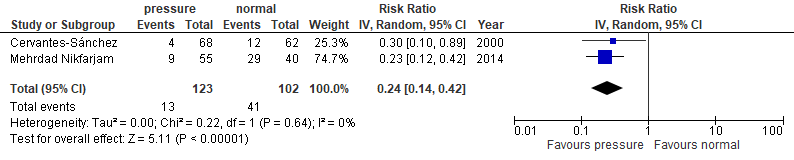


**Fig 5-7: Forest plot of the meta-analysis of high-pressure wound irrigation (observational studies).**


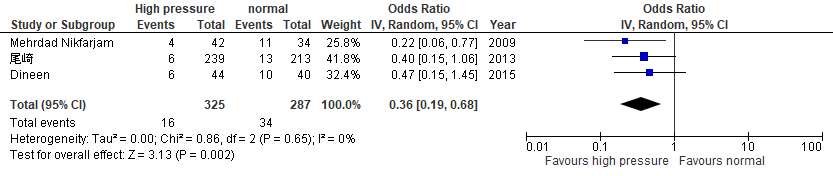


Ozaki

**Fig 5-8. Forest plot of the meta-analysis of peritoneal lavage (RCTs)**


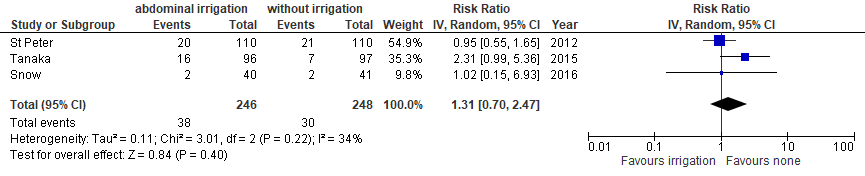


**Fig. 5-9. Forest plot of the meta-analysis of peritoneal lavage (observational studies).**


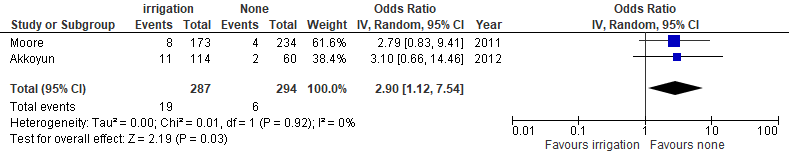


**Fig. 5-10. Mortality rate within 30 days after total gastrectomy according to the use of a drain versus no drain**


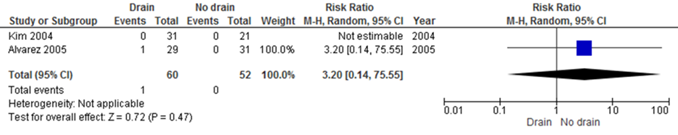


**Fig. 5-11. Mortality rate within 30 days after distal gastrectomy according to the use of a drain versus no drain**


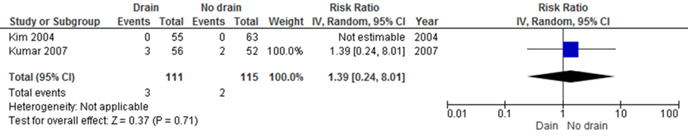


**Fig. 5-12. SSI rate after laparoscopic cholecystectomy according to the use of a drain versus no drain**


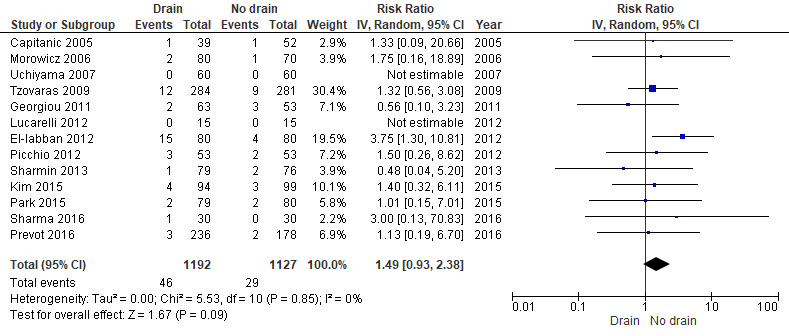


**Fig. 5-13. Operation time after laparoscopic cholecystectomy according to the use of a drain versus no drain**


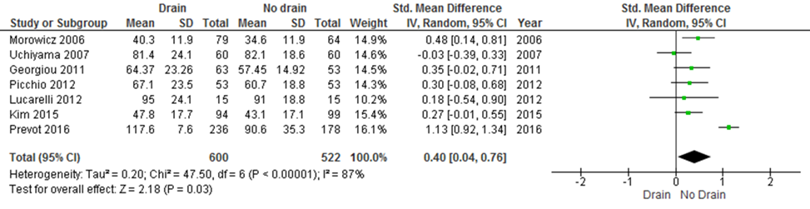


**Fig. 5-14. Mortality rates after hepatectomy according to the use of a drain versus no drain**


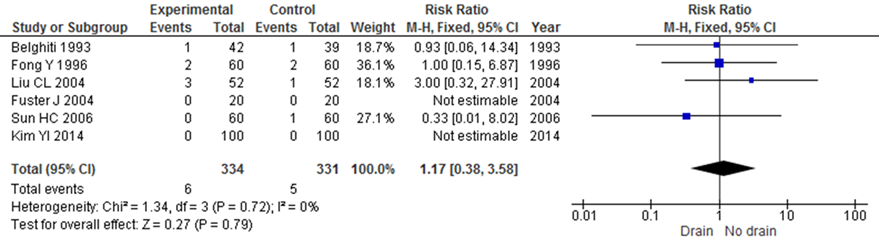


**Fig. 5-15. SSI rates after hepatectomy according to the use of a drain versus no drain**


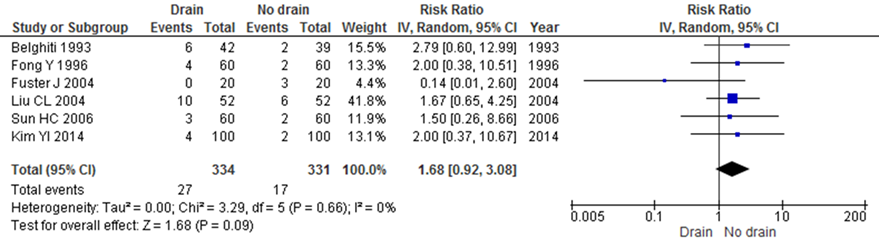


**Fig. 5-16. Ascitic leakage after hepatectomy according to the use of a drain versus no drain**


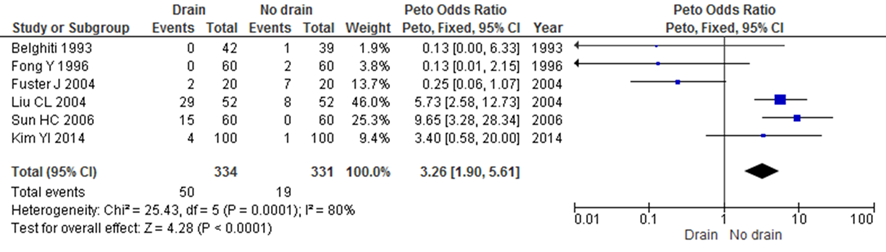


**Fig. 5-17. Mortality rates after pancreatoduodenectomy according to the use of a drain versus no drain**


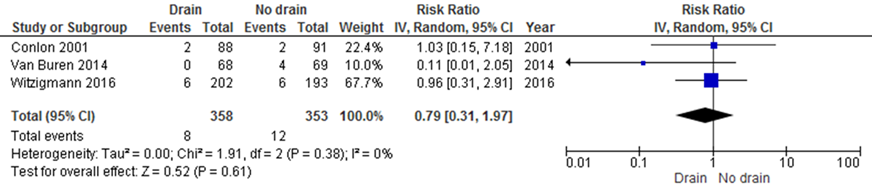


**Fig. 5-18. Intraabdominal infection following pancreatoduodenectomy after early versus late drain removal**


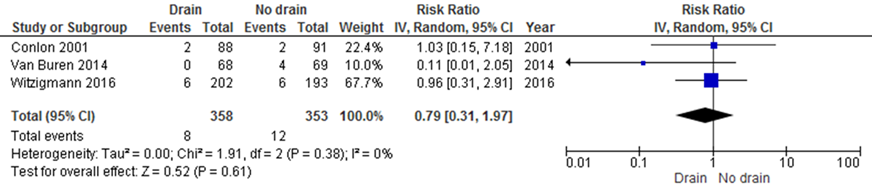


**Fig. 5-19. Hospital stay following pancreatoduodenectomy after early versus late drain removal**


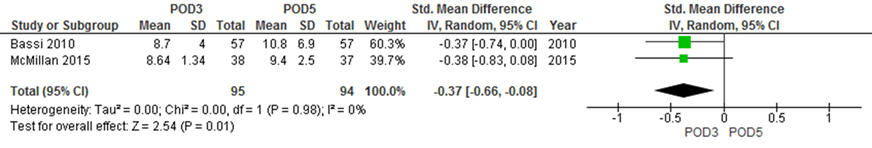


**Fig. 5-20. Mortality rate after appendectomy with versus without a drain**


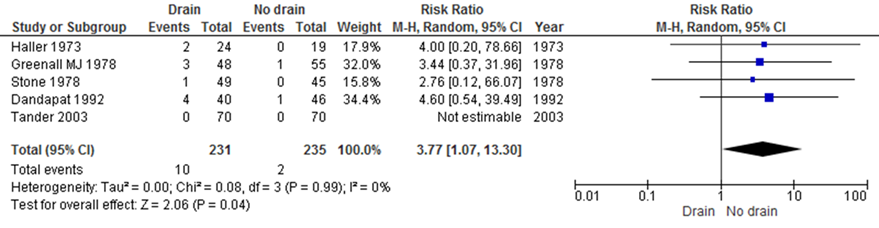


**Fig. 5-21. Drain versus no-drain, outcome: Mortality rate after rectal surgery with versus without a drain**


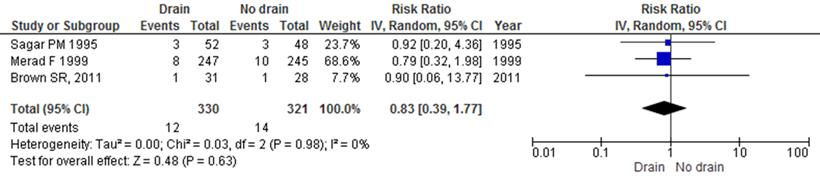


**Fig. 5-22. SSI rate after general surgery with versus without a subcutaneous drain**


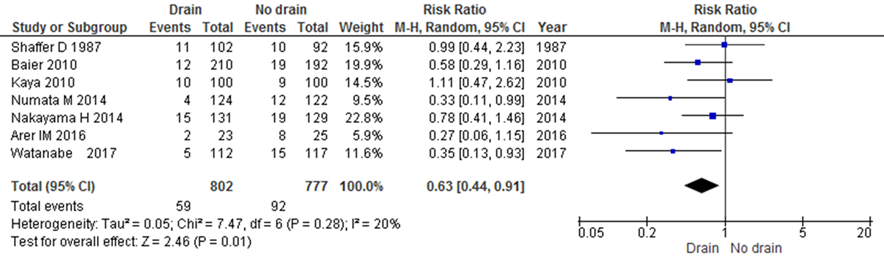


**Fig. 5-23. SSI rate after general surgery with absorbable versus non- absorbable sutures**


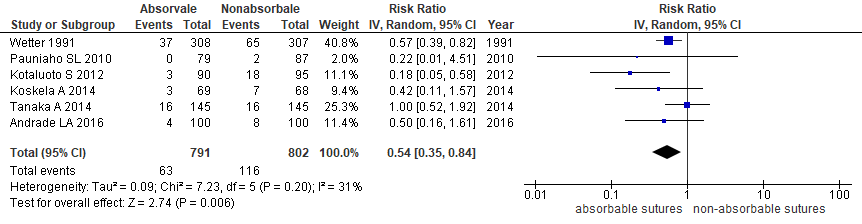


**Fig. 5-24. Wound dehiscence associated with continuous sutures versus interrupted sutures**


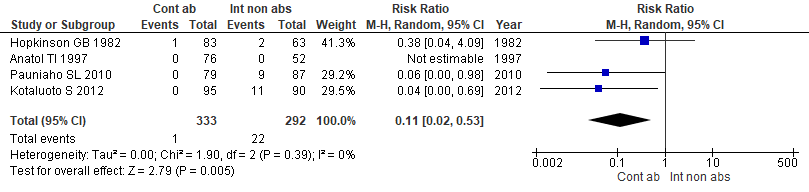


**Chapter 6. Perioperative management**

**Fig. 6-1. SSI incidence with ERP versus conventional management**

**Fig. 6-2. SSI incidence with preoperative CHO versus a placebo**

**Fig. 6-3. SSI incidence with intensive glucose control versus conventional control SSI**

**Fig. 6-4. Incidence of pneumonia with vs. without intraoperative oral hygiene**

**Fig. 6-5. SSI incidence associated with intraoperative warming**


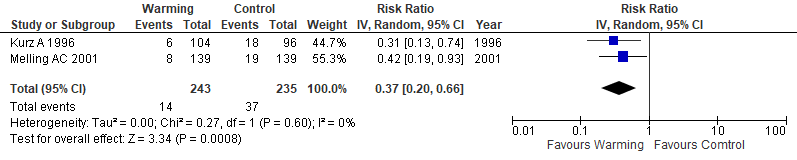


**Fig. 6-6. SSI incidence associated with high perioperative F_I_O_2_ in digestive surgery**


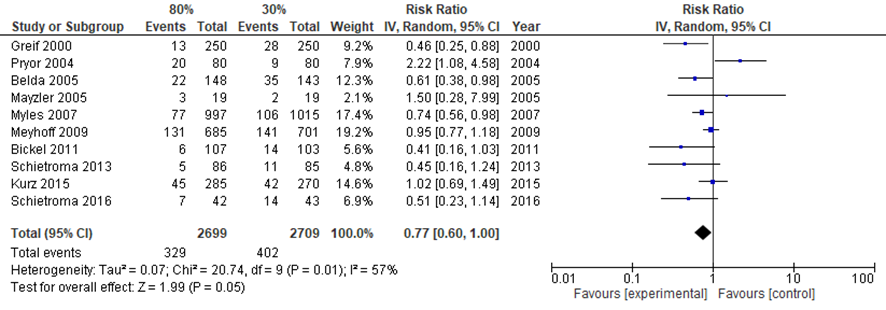


**Fig. 6-7. SSI incidence associated with high F_I_O_2_ during and 2–6 hours after colorectal surgery**


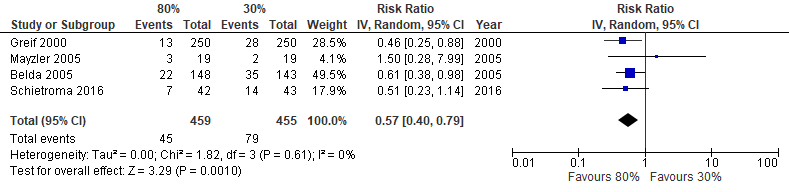


**Fig. 6-8. SSI incidence after laparoscopic cholecystectomy with early postoperative oral and enteral feeding versus conventional feeding**


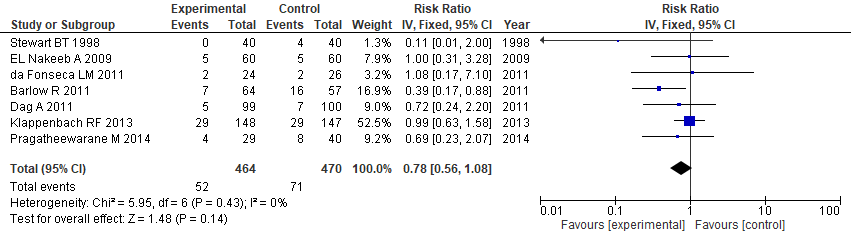


**Chapter 7. Wound management**

**Fig. 7-1. SSI incidence after general surgery with a protective wound dressing versus a gauze dressing**


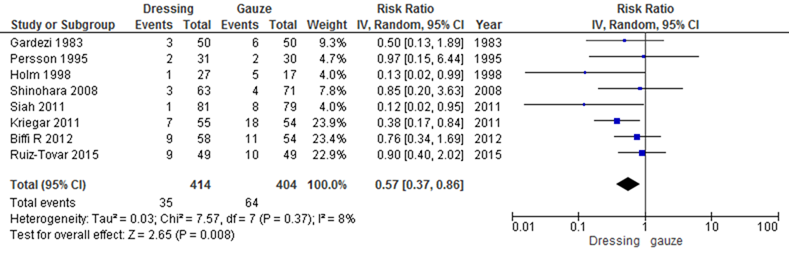


**Fig. 7-2. SSI of the primary incisional wound after NPWT versus standard management**


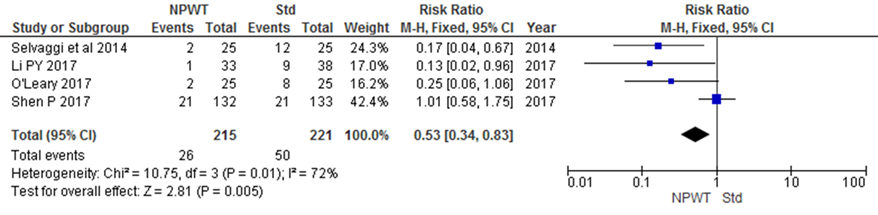


**Fig. 7-3. Seroma formation of the primary incisional wound after NPWT versus standard management**


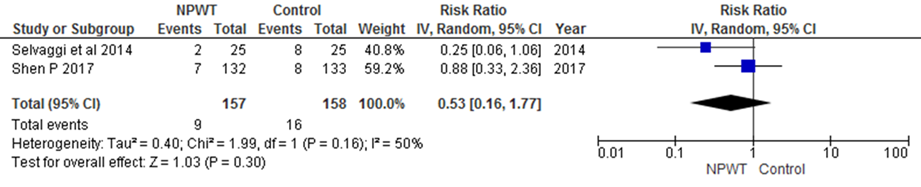

Supplement: Supplementary file 1 — Supplementary file1. (DOCX 2281 kb) [file 595_2020_2181_MOESM1_ESM.docx]
